# Optimizing early-phase immunotherapy trials: the role of biomarker enrichment strategies

**DOI:** 10.3389/fimmu.2025.1664443

**Published:** 2025-10-22

**Authors:** Greta Catani, Daniel Morchón-Araujo, Oriol Mirallas, Vicky Sánchez-Pérez, Paolo Nuciforo, Guillermo Villacampa, Rodrigo Dienstmann, Ana Vivancos, Elena Garralda, Alberto Hernando-Calvo

**Affiliations:** ^1^ Department of Medical Oncology, Alexander Fleming Institute, Buenos Aires, Argentina; ^2^ Vall d´Hebron Institute of Oncology (VHIO), Barcelona, Spain; ^3^ Department of Medical Oncology, University Hospital of Salamanca, IBSAL, Salamanca, Spain; ^4^ Department of Medical Oncology, Vall d´Hebron Barcelona Hospital Campus, Barcelona, Spain; ^5^ Departament de Medicina, Universitat Autònoma de Barcelona, Barcelona, Spain; ^6^ Molecular Oncology Group, Vall d´Hebron Institute of Oncology, Barcelona, Spain; ^7^ Oncology Data Science, Vall d´Hebron Institute of Oncology (VHIO), Barcelona, Spain; ^8^ University of Vic, Central University of Catalonia, Vic, Spain; ^9^ Oncoclinicas Medicina de Precisão, Oncoclínicas & Co, São Paulo, Brazil

**Keywords:** biomarker, immune checkpoint inhibitors, early-phase trials, phase 1, immunotherapy, drug response biomarkers, predictive biomarkers

## Abstract

Immune checkpoint inhibitors have revolutionized the treatment of solid tumors; however, their clinical efficacy remains limited to a subset of patients. Novel immunotherapy agents are being investigated in phase I clinical trials, with an increasing focus on biomarker selection strategies to optimize patient outcomes. Prior evidence suggests that biomarker-selected tumors may have better outcomes when treated with molecularly-guided therapies. However, the high complexity of tumor-host interactions and inter-patient variability indicates that a one-size-fits-all biomarker approach is unlikely to be sufficient in the immunotherapy landscape. This review highlights current biomarker-enrichment strategies in immunotherapy early drug development, addressing challenges and potential future directions for their effective implementation.

## 1 Introduction

Immune checkpoint inhibitors (ICIs) have dramatically changed the therapeutic landscape of several tumor types, offering durable responses, prolonged survival, and even the potential for long-term remission in a small subset of patients. The approval of ipilimumab, the first anti-CTLA-4 monoclonal antibody (mAb), for the treatment of melanoma in 2011 marked the advent of a new era (1). This was followed by significant advances with the development of anti-programmed cell death 1 (anti-PD-1) or its ligand (anti-PD-L1) mAb, such as nivolumab or pembrolizumab, becoming part of the standard treatment for multiple cancer types (2–4). Despite these therapeutic milestones, nearly 80% of patients either fail to respond to ICIs or develop resistance over time (5). Furthermore, a clinically significant proportion of patients treated with ICIs experience severe immune-related adverse events (irAEs), which lead to treatment discontinuation, and a subset may even exhibit rapid disease progression, a phenomenon known as hyperprogression (6–8). To address current limitations with classic ICIs, an expanding array of next-generation immunotherapeutic agents—including novel checkpoint inhibitors, T-cell engagers, cytokine modulators, and cell-based therapies—is being evaluated in early-phase trials, aiming to broaden the population of responders while overcoming mechanisms of intrinsic or delaying acquired resistance (9, 10). A critical priority in immunotherapy early drug development is optimizing patient selection to accelerate efficacy signals while mitigating toxicity risks, even during dose escalation phases (11).

Currently, fewer than 10% of oncology drugs entering phase 1 trials ultimately receive regulatory approval (12). This rate may be even lower for immunotherapies due to their biological and clinical complexities, underscoring a critical need, as the development of immunotherapy agents is a highly complex, time-intensive, and costly process (13). A pivotal shift in immuno-oncology (IO) has been the growing emphasis on biomarker enrichment strategies. A comprehensive analysis, which reviewed 17,368 drug development trajectories (2000-2015), found biomarker-guided patient selection significantly increased success rates in drug development (10.7% vs 1.6%) (12). Similarly, the use of biomarker-guided therapies has been associated with better outcomes in patients treated with immunotherapy in phase 1–2 trials (14). Overall, these data highlight the need to implement biomarker-driven enrichment strategies in early-phase immunotherapy trials to refine patient selection and enhance trial efficiency. In this regard, the tumor-agnostic approval of pembrolizumab in 2017 for the treatment of microsatellite instability-high (MSI-H) or mismatch repair–deficient (dMMR) tumors represented a paradigm shift. This marked the first time the U.S. Food and Drug Administration (FDA) approved a cancer therapy based on the presence of a molecular biomarker independently of tumor histology (15, 16). Moreover, tumor mutational burden (TMB) has also emerged as another critical predictive biomarker. TMB high (TMB-H), defined by the presence of equal or more than ten mutations per megabase (Mb), has been correlated with increased neoantigen load and improved responses to ICIs across various malignancies (17, 18). Indeed, the FDA approved TMB in 2020 as a tissue-agnostic companion diagnostic for pembrolizumab in solid tumors, based on the results of the KEYNOTE-158 phase 2 trial, which showed an overall response rate (ORR) of 29% (19, 20). Furthermore, to optimize novel drug development, biomarker discovery and validation have been increasingly shifting from late-stage studies to early-phase clinical trials. However, despite substantial efforts in searching robust biomarkers, only programmed death-ligand 1 (PD-L1) expression, TMB, and dMMR/MSI-H are currently validated and used in routine clinical practice, while others remain under investigation (**Table 1**) (35). Integrating high-throughput technologies into early-phase clinical trial design may improve efficiency, patient selection and refine endpoint determination (36).

**Table 1:** Established and emerging biomarkers for immunotherapy in solid tumors.

Biomarker	Predictive value	Assay/Tissue type	Limitations
PD-L1 (21)	Predicts response in NSCLC, HNSCC, gastric, TNBC, cervical, urothelial	IHC (clones: 22C3, 28-8, SP142, SP263) on tumor and/or immune cells	- Heterogeneity (intra/inter-tumoral, temporal). - Assay variability. - Lack of universal cut-off. - Variability in predictive value across tumor types.
dMMR/MSI-H (22, 23)	Strong predictor of response; FDA-approved for pembrolizumab (tissue-agnostic)	IHC (MLH1, MSH2, MSH6, PMS2); PCR; NGS	- Discordant cases (IHC vs PCR/NGS) may still respond. - Biological heterogeneity (co-alterations may modulate response).
TMB-H (17, 18)	Predicts response; FDA-approved for pembrolizumab (tissue-agnostic)	Targeted panels, WES, WGS or liquid biopsy (ctDNA)	- Lack of standardization. - Variable panel sizes. - Predictive value inconsistent across tumor types.
*POLE/POLD1* mutations in EDMs (24)	Predict sensitivity (ultramutator phenotype)	NGS, Sanger sequencing, FFPE assays	- Very low prevalence (<3%). - Requires broad prescreening.
SWI/SNF complex (ARID1A, PBRM1, SMARCA4, SMARCB1) (8)	Predict potential sensitivity	NGS, molecular profiling	- Exploratory.
Resistance mutations (*B2M, JAK1/2, STK11/LKB1, KEAP1, EGFR, PTEN, MDM2*) (7, 8, 25–34)	Associated with resistance or hyperprogression	NGS, molecular profiling	- Context-dependent: not absolute (e.g., some MSI-H with B2M/JAK1/2 still respond). - Remain exploratory.

NSCLC, non-small cell lung cancer; HNSCC, neck squamous cell carcinoma; TNBC, triple-negative breast cancer; IHC, immunohistochemistry; TPS, Tumor Proportion Score; CPS, Combined Positive Score; ctDNA, circulating tumor DNA; PCR, polymerase chain reaction; NGS, next-generation sequencing; WES, Whole exome sequencing; WGS, Whole genome sequencing; FFPE, specialized mutation assays using formalin-fixed paraffin-embedded; EDMs, exonuclease domains.

In this narrative review, we aimed to provide a comprehensive overview of the current landscape of biomarkers and enrichment strategies in immunotherapy while highlighting advances, limitations, and persistent barriers associated with the broad implementation of biomarker-driven patient selection in early-phase trials.

## 2 Tissue-based immunotherapy biomarkers

### 2.1 Programmed cell death-ligand 1

PD-L1 has been extensively investigated for predicting response to ICIs (37). PD-L1 immunohistochemistry (IHC) has received regulatory approval as a companion or complementary diagnostic test for several ICIs across various tumor types, including non-small cell lung cancer (NSCLC), gastric cancer, triple-negative breast cancer (TNBC), cervical cancer, urothelial carcinoma, and head and neck squamous cell carcinoma (HNSCC) (21). Despite its initial promise, translating it into a consistently reliable biomarker for patient selection has been significantly challenged by inherent biological complexities and technical standardization (38). PD-L1 expression can be induced by cytokines such as interferon-gamma (IFN-γ), exhibiting notable intra-tumoral and inter-metastatic heterogeneity, and frequently diverges between primary tumors and metastases (39–41). For example, discordant expression was observed in 50% of advanced melanoma patients when they were longitudinally sampled (40). Also, PD-L1 expression can be modulated by prior or concurrent treatments, adding temporal variability as another layer of complexity (41–43). For instance, in a cohort of patients with metastatic NSCLC, prior ICI exposure was associated with reduced PD-L1 expression compared to treatment-naïve patients (44). This results in significant heterogeneity within and across tumor sites, making a single pre-treatment biopsy a potentially unreliable indicator of the overall immune landscape (19, 20, 45). Moreover, the predictive value of PD-L1 varies depending on whether expression is assessed on tumor cells (TCs) or immune cells (ICs). While PD-L1 expression on TCs has shown a more consistent correlation with treatment outcomes, expression on ICs has demonstrated variable and often limited associations with response to ICIs (39). Such sampling bias can lead to misclassification, limiting the ability to detect efficacy signals, which is especially critical in small, exploratory phase 1 trials. PD-L1 expression can be evaluated using different scoring systems, such as the Tumor Proportion Score (TPS) and the Combined Positive Score (CPS), each with distinct clinical cut-offs specific to the assay, tumor type, and therapeutic agent. Moreover, differential predictive value has been observed across these methods, adding further complexity to the interpretation of PD-L1 expression (21). Beyond biological complexity, the utility of PD-L1 as a reliable biomarker is further constrained by significant technical challenges. The FDA has approved multiple IHC assays for PD-L1 detection utilizing different antibody clones (Dako 22C3, Dako 28-8, Ventana SP142, and Ventana SP263), each developed in conjunction with specific PD-(L)1 inhibitors (21).

In phase 1 clinical trials, the use of different thresholds to define PD-L1 positivity has yielded variable results, underscoring the lack of standardization in the application of this biomarker. The expansion cohort of the KEYNOTE-001 reported an ORR of 45%, 16.5% and 10.7% in patients with a TPS ≥50%, 1–49% and <1%, respectively (46). These findings led to FDA approval for PD-L1 expression and provided the basis for subsequent trials such as KEYNOTE-010 (cut-off ≥1%) and KEYNOTE-024 (cut-off ≥50%) in NSCLC (4, 47). Similarly, other anti-PD-(L)1 mAbs (e.g., atezolizumab or nivolumab) demonstrated responses predominantly in patients with high PD-L1 expression (**Table 2**) (48, 59). However, growing evidence suggests that PD-(L)1 blockade efficacy may be independent of PD-L1 expression in specific contexts, with clinical benefits observed in PD-L1 negative tumors (2, 60). For example, in advanced renal cell carcinoma (RCC) treated with nivolumab, the median overall survival (OS) was paradoxically longer in the PD-L1<1% population (27.4 months) compared to PD-L1≥1% group (21.8 months), challenging the pan-cancer predictive value (61). Overall, these limitations constrain its pan-cancer application. While PD-L1 remains an established biomarker in clinical decision-making for approved ICIs in specific, well-defined contexts, its profound biological dynamism and susceptibility to technical variability render it an insufficient enrichment tool in early-phase drug development (62). Assay-dependent misclassification and arbitrary thresholds may overestimate drug activity in biomarker-selected populations or inadvertently exclude PD-L1–negative patients who could benefit from treatment. Therefore, while PD-L1 retains clinical utility as an accessible and widely implemented biomarker, particularly for patient selection in specific indications, its limitations suggest that it may be more appropriately integrated as a stratification factor, an exploratory endpoint, or as part of a more comprehensive, multi-parametric biomarker approach.

**Table 2:** Main diagnostic assays and thresholds enabling ICI use in common solid tumors.

Tumor type	Approved biomarker	Assay/Clone	FDA-approved threshold for ICI use	Reference trial
NSCLC	PD-L1 (TPS)	IHC (22C3, 28-8, SP263)	TPS ≥50%: pembrolizumab (4) TPS ≥1%: pembrolizumab (47) or nivolumab plus ipilimumab (48)	KEYNOTE-024 (4), KEYNOTE-010 (47), CheckMate-227 (48)
HNSCC	PD-L1 (CPS)	IHC (22C3)	CPS ≥1: pembrolizumab (49)	KEYNOTE-048 (49)
Gastric/GEJ	PD-L1 (CPS)	IHC (22C3)	CPS ≥5: nivolumab (50) CPS ≥10: pembrolizumab (51)	CheckMate 649 (50) KEYNOTE-859 (51)
Colorectal Cancer	dMMR/MSI-H	IHC (MLH1, MSH2, MSH6, PMS2); PCR; NGS	dMMR/MSI-H	KEYNOTE-177 (52), CheckMate-8HW (53)
Cervical cancer	PD-L1 (CPS)	IHC (22C3)	CPS ≥1: pembrolizumab (54)	KEYNOTE-826 (54)
Urothelial carcinoma	PD-L1 (CPS or IC%)	IHC (22C3, SP142)	CPS ≥10: pembrolizumab (55) IC ≥5%: atezolizumab (56)	IMvigor210 (56), KEYNOTE-052 (55)
Tissue-agnostic	dMMR/MSI-H	IHC (MLH1, MSH2, MSH6, PMS2); PCR; NGS	dMMR/MSI-H (any tumor)	KEYNOTE-016 (57), KEYNOTE-158 (58)
Tissue-agnostic	TMB-H	NGS (FoundationOne CDx)	≥10 mut/Mb	KEYNOTE-158 (58)

NSCLC, non-small cell lung cancer; IHC, immunohistochemistry; TPS, Tumor Proportion Score; HNSCC, neck squamous cell carcinoma; CPS, Combined Positive Score; GEJ, gastroesophageal junction; IC, immune cells; NGS, next-generation sequencing; Mb, megabase.

### 2.2 Mismatch repair deficiency

Tumors harboring deficiencies in the DNA mismatch repair (MMR) pathway characteristically accumulate an exceptionally high number of somatic mutations, a state known as MSI-H. This high mutational load makes dMMR/MSI-H tumors particularly rich in immunogenic neoantigens, providing a strong rationale for their generally higher sensitivity to ICIs (22, 23). The prevalence of dMMR/MSI-H tumors varies across cancer types, ranging from approximately 30% of the cases in endometrial cancer (EC), 10-15% in colorectal cancer (CRC), 5-8% in gastric cancers, and under 5% in many other solid tumors (63). Detection methods include IHC for MMR proteins (MLH1, MSH2, MSH6, PMS2), as well as molecular techniques such as polymerase chain reaction (PCR) and next-generation sequencing (NGS) to assess MSI status (**Table 1**) (64). While NGS and PCR concordance is generally high, with a reported rate of approximately 98.8%, historical discordance rates between IHC and molecular methods (NGS/PCR) have ranged from 1-10% (64, 65). More recent data using improved methodologies suggest a lower discordance rate (0.3–1.6%) (**Supplementary Table S1**) (66, 67). However, each technique offers unique strengths and limitations. While IHC offers shorter turnaround times and cost-effectiveness, it may fail to identify dMMR in cases of missense mutations where non-functional proteins retain antigenicity. Conversely, NGS provides superior sensitivity, detecting MSI-H tumors that are missed by IHC (e.g., those with atypical gene involvement as MLH1, PMS1, or co-occurring *POLE* mutations), but it often entails longer turnaround time and higher costs (68, 69). Importantly, discordant cases such as pMMR/MSI-H tumors may still harbor a high mutational burden and consequently respond favorably to ICIs (17, 67). Therefore, the choice of detection method in early-phase trials may be significant, as it can influence the characterization of the enriched patient population and the subsequent interpretation of efficacy signals (63).

The clinical utility of dMMR/MSI-H as a predictive biomarker for ICIs was first demonstrated across multiple studies. KEYNOTE-016, a phase 2 study, reported an ORR of 40% and 71% with pembrolizumab in advanced dMMR/MSI-H CRC and dMMR/MSI-H non-CRC, respectively (57). Subsequently, the phase 2 multicohort trial, KEYNOTE-158, showed an ORR of 34.3% with a median progression-free survival (PFS) of 4.1 months and OS of 23.5 months, in pre-treated patients with various dMMR/MSI-H non-CRC solid tumors (58). These findings culminated in the 2017 FDA accelerated approval of pembrolizumab for dMMR/MSI-H solid tumors, establishing it as the first tissue-agnostic biomarker for cancer therapy (**Table 2**) (70). More recently, phase 3 trials, such as CheckMate 8HW and KEYNOTE-177, have confirmed the benefit of ICIs in the first-line treatment of dMMR/MSI-H mCRC compared with standard chemotherapy combinations (52, 53).

Despite robust evidence, there is a critical need for further understanding of dMMR/MSI-H tumors. Not all dMMR/MSI-H tumors respond equally, suggesting biological heterogeneity that could guide future enrichment strategies. For example, the presence of co-occurring oncogenic alterations in the *PI3K-AKT* signaling pathway (e.g., *PTEN* loss, *AKT1* mutations) has been implicated in primary resistance to ICIs. Such alterations are hypothesized to contribute to an immune-excluded phenotype by modulating cytokine dysregulation, reducing T-cell infiltration, and impairing antigen presentation (71). Interestingly, loss of expression of both *MLH1* and *PMS2* has been associated with stronger responses due to greater neoantigen load (71). Additionally, discordant profiles, such as pMMR/MSI-H tumors, may still exhibit TMB-H and respond favorably to ICIs (17, 67). These data suggest that further patient stratification within the dMMR/MSI-H population may be required. Early-phase trials could be designed to identify these “hyper-responders” subgroups or rationally design combination therapies aimed at overcoming these potentially resistant pathways.

### 2.3 Tumor mutational burden

TMB is defined as the total number of non-synonymous mutations per Mb of the tumor genome and can be considered as a surrogate marker of overall tumor immunogenicity, emerging as a potential pan-cancer predictive biomarker for response to ICIs. TMB can be estimated using NGS approaches, ranging from whole-genome/exome sequencing (WGS/WES) of paired tumor and normal DNA to targeted gene panels based on tumor tissue or liquid biopsies (68, 69). However, its clinical application is hindered by a lack of standardization across testing platforms and analytical methodologies. We summarized common assays used in clinical practice in **Supplementary Table S2**. WES-based TMB calculations typically focus on missense mutations, but TMB values are also highly influenced by pre-analytical factors such as tumor purity, with low purity potentially leading to TMB underestimation (72, 73). Moreover, variability in panel size, sequencing depth, and mutation filtering criteria further complicates cross-platform comparability and limits the utility of TMB for prospective patient selection in early-phase trials (74). International initiatives such as Friends of Cancer Research (Friends) and the Quality in Pathology (QuIP) have developed frameworks aimed at standardizing and harmonizing TMB assessment across platforms and centers globally (75, 76).

Based on the results of the single-arm phase 2 KEYNOTE-158 trial showing an ORR of 29% across tumor histologies, the FDA granted the tumor-agnostic approval of pembrolizumab for patients with unresectable or metastatic solid tumors exhibiting TMB ≥10 mutations/Mb, as determined by a specific assay (FoundationOne CDx) (**Tables 1**, **2**) (19). Further evidence has emerged from clinical trials in gastric cancer (such as KEYNOTE-061), HNSCC (CONDOR and HAWK), or hepatocellular carcinoma (EPOC1704), which have contributed to this evidence base, particularly for combination immunotherapy strategies (77–82). Another study found that higher log-transformed TMB values were associated with improved response rates. Notably, some viral-related cancers (anal, cervical, and hepatocellular) exhibited better responses than expected based solely on TMB, suggesting that additional factors may influence ICI responses (83). While this approval marked a significant step forward in precision IO, major concerns still exist around the clinical application of TMB. For instance, the predictive value of TMB is not consistent across tumor types. Despite the presence of TMB-H in certain tumors such as breast, prostate cancer, and gliomas, TMB has not been definitively associated with increased immunotherapy sensitivity. This suggests that a TMB-H is not necessarily indicative of effective immune recognition, potentially due to factors such as a lack of functional neoantigens or insufficient CD8+ T-cell infiltration (84). Importantly, the predictive value is not universal, so its utility depends significantly on tumor-intrinsic and patient-specific characteristics. For instance, a retrospective study analyzing survival outcomes in over 8000 patients with solid tumors treated with ICIs showed that TMB-H was significantly associated with longer OS in the pan-cancer setting, including the CRC subgroup. Nevertheless, among patients with MSS CRC, no differences were observed according to TMB status, suggesting that this benefit may be driven by the dMMR/MSI-H sub-population (85).

Moreover, in a cohort of 1,662 patients analyzed using the MSK-IMPACT targeted NGS panel, higher somatic TMB (defined as the top 20% within each histologic subtype) was associated with improved OS with ICIs across cancer types. However, specific TMB cut-offs defining this “high” stratum varied markedly between histologies, suggesting a universal TMB threshold is unlikely to be optimal (86). Commonly used assays for TMB evaluation are presented in **Supplementary Table S2**. Consequently, applying fixed TMB thresholds for patient enrichment in early drug development, particularly across heterogeneous pan-tumor cohorts, risks oversimplification. This could compromise sensitivity by excluding potential responders or reduce specificity by including non-responsive TMB-H individuals. Notably, a lack of concordance has been observed between TMB and other biomarkers such as PD-L1 expression. For instance, in CheckMate-026 and CheckMate-227, patients with NSCLC who had TMB-H experienced prolonged PFS with ICI treatment, regardless of PD-L1 status (87, 88). This supports the idea that TMB may define a distinct subset of immunotherapy-responsive patients, underscoring the need for integrative, multi-parametric biomarker strategies in early-phase settings. Recognizing the limitations of tissue-based TMB (tTMB) assessment, plasma-based TMB (blood TMB or bTMB) offers a less invasive alternative. Clinical trials, such as MYSTIC and BFAST, in NSCLC have explored bTMB, demonstrating its feasibility, as well as the challenges, including assay failure due to insufficient circulating tumor DNA (ctDNA) (25-30% in MYSTIC) and only moderate concordance (around 50-60%) with tTMB (89, 90). Currently, the clinical implementation of bTMB remains constrained by technical and biological limitations. These include but are not limited to low ctDNA abundance in early-stage disease, low-shedding tumors, and the resulting suboptimal sensitivity and concordance with tTMB (91). In summary, although TMB shows potential as a predictive biomarker for ICIs response, its clinical utility remains constrained by methodological variability and the need for integration with complementary biomarkers.

### 2.4 Genomic determinants of immunotherapy sensitivity and resistance

Specific genomic alterations may indicate increased sensitivity or inherent resistance to immunotherapy and may have a role in guiding patient stratification and rational combination approaches in early-phase clinical trials. Pathogenic mutations in the exonuclease domains (EDMs) of DNA polymerases epsilon (POLE) and delta 1 (POLD1) are emerging as predictive biomarkers of sensitivity to ICIs. These mutations impair proofreading, leading to an “ultramutator” phenotype characterized by a high frequency of base substitution mutations, distinct from the indel-driven frameshifts common in MSI-H tumors (**Table 1**) (24). Somatic or germline *POLE/POLD1* mutations in EDMs occur at low frequencies; somatic *POLE* and *POLD1* mutations were estimated at 2.79% and 1.37%, respectively (92, 93). Notably, *POLE* EDMs and dMMR/MSI-H can co-occur, potentially creating an exceptionally immunogenic neoantigen landscape enriched by MSI-H-driven indels, which could enhance T-cell infiltration (93). Promising clinical evidence supports their predictive value. For instance, a phase 2 trial evaluating the anti-PD-1 agent, toripalimab, in advanced solid tumors, reported an ORR of 21.4%, with a notably higher response rate among patients harboring *POLE* EDMs (66.7%) compared to those with non-EDM *POLE/POLD1* variants (9.1%) (94). Similarly, the KEYNOTE-028 study reported durable responses to pembrolizumab in patients with *POLE*-mutated EC (95). Prospective studies are ongoing (e.g., NCT05103969, NCT02693535, NCT03491345, and NCT06118658) (96–98). Despite their distinct biology and potential for profound ICI sensitivity, their low pan-cancer incidence poses a practical hurdle for patient enrichment in early-phase trials, often requiring extensive molecular prescreening programs or a strategic focus on tumor types with already known higher prevalence, like EC. Alterations in chromatin remodeling complexes, particularly components of the SWI/SNF complex (e.g., ARID1A, PBRM1, SMARCA4, SMARCB1), have also emerged as potential biomarkers of ICI sensitivity, outlined in **Table 1** (8). These mutations can lead to increased mutational burden, an inflamed tumor microenvironment (TME), and enhanced immune infiltration, suggesting their utility in identifying responsive subgroups across various tumor types (8, 99–101).

Conversely, multiple genomic alterations have been implicated in both primary and acquired resistance to ICIs. Distinct but functionally convergent mechanisms of immune resistance include alterations in β2-microglobulin (B2M), which impair antigen presentation by disrupting MHC-I; inactivating mutations in *JAK1/2*, key mediators of IFN-γ signaling; and chromosomal losses like 9p21.3, which encompass *CDKN2A/B* and potentially *JAK2*, leading to immune exclusion and reduced CD8+ T cell infiltration (8, 25, 26, 102). The close genomic proximity of *CDKN2A* and *JAK2* on chromosome 9p facilitates frequent co-deletions, which have been observed across multiple tumor types, including melanoma (75%), lung squamous cell carcinoma (90.5%), and bladder urothelial carcinoma (80%) (103). These co-deletions are associated with impaired IFN-γ signaling, diminished immune infiltration, and further diminishing ICI sensitivity (26, 102–106). Similarly, loss-of-function mutations in *B2M* or *JAK1/2* have been identified in patients with melanoma that progresses on anti–PD–1 therapy, suggesting acquired resistance, **Table 1** (8, 25, 26). However, emerging evidence indicates that the presence of these mutations does not uniformly predict treatment failure. For instance, among patients with dMMR/MSI-H CRC, the presence of *B2M* or *JAK1/2* mutations associated with clinically meaningful responses to PD-1 blockade, challenging the assumption that these alterations universally mediate resistance (107). Inactivation of the tumor suppressor *Serine/Threonine kinase 11* (*STK11/LKB1*), frequently observed in *KRAS*-mutant NSCLC, is strongly associated with primary resistance to ICIs, even in tumors with TMB-H (27, 28). This alteration contributes to an immunologically “cold” TME, characterized by poor T-cell infiltration and low PD-L1 expression (27). Similarly, loss-of-function mutations in *KEAP1*, which lead to constitutive activation of the NRF2 pathway, are associated with immune exclusion and ICI resistance (29). *KEAP1* mutations occur in approximately 2.7% of all cancers and are particularly enriched in NSCLC (15.8%) (8). *EGFR* mutations, also common in NSCLC, may promote immune escape through the upregulation of PD-(L)1 and CTLA-4, and *EGFR* amplifications have been implicated in hyperprogression following ICI therapy. However, supporting data remain inconsistent (7). In addition, *PTEN* loss fosters an immunosuppressive TME via activation of the *PI3K–AKT* signaling pathway, while *MDM2* amplification, potentially through NFATc2 degradation, has also been associated with hyperprogression and resistance (30–34). Emerging genomic alterations that mediate resistance or hyperprogression under ICI therapy are summarized in **Table 1**. Together, these alterations outline a molecular landscape of intrinsic resistance to immunotherapy. Understanding this landscape of resistance mutations is critical for refining patient selection in early-phase clinical trials, potentially by excluding patients unlikely to benefit from certain monotherapies or by identifying candidates for rational combination approaches designed to overcome these specific resistance mechanisms.

## 3 Non-invasive immunotherapy biomarkers

### 3.1 Circulating tumor DNA

While profiling specific genomic alterations and other tissue-based biomarkers provides critical biological insights, the static nature of single biopsy timepoints limits this approach. ctDNA has emerged as a minimally invasive biomarker offering dynamic insights into tumor burden, mutational landscapes and therapeutic response. ctDNA encompasses fragmented DNA released into the bloodstream from apoptotic or necrotic tumor cells, circulating tumor cells, and tumor-derived exosomes. Its potential to revolutionize early-phase immunotherapy trials lies in its ability to provide real-time molecular data, overcoming many limitations of traditional tissue-based tumor biopsies. Several studies support the prognostic and monitoring utility of ctDNA in patients receiving ICIs. Persistently detectable ctDNA levels during anti-PD-(L)1 treatment have been associated with worse clinical outcomes (108–110). For example, a prospective phase 2 trial (NCT02644369) evaluated patients with advanced solid tumors treated with pembrolizumab. The study found that a decrease in ctDNA levels from baseline was associated with better outcomes under immune checkpoint blockade (111). These findings have been supported by additional pieces of data across multiple tumor types, highlighting the potential of ctDNA early dynamics to serve as a prompt surrogate endpoint for ICI efficacy outcomes (112).

Compared to conventional tissue-based molecular profiling, ctDNA offers distinct advantages. For instance, it is minimally invasive, facilitating repeated sampling for longitudinal monitoring; it may more accurately reflect spatial and temporal tumor heterogeneity; and it may be able to detect earlier response or resistance compared to standard imaging (113, 114). These attributes are particularly valuable in early-phase immunotherapy trials, where timely and dynamic genomic insights can inform patient selection, cohort stratification, and early assessment of treatment response (115). Importantly, ctDNA also enables the non-invasive evaluation of key immunotherapy biomarkers such as TMB and MSI. Several commercial and academic platforms have demonstrated the feasibility of estimating bTMB and MSI status from ctDNA (116). However, as discussed previously, limitations in analytical sensitivity, particularly in patients with low tumor burden or low ctDNA shedding, remain a challenge (117). Substantial heterogeneity currently exists across ctDNA testing platforms. Differences in laboratory-developed protocols, sequencing technologies, bioinformatic pipelines, and variant calling thresholds can lead to discordant findings, posing challenges for assay reproducibility and clinical interpretation (117). Successful integration of ctDNA into routine clinical trials and practice would benefit from standardized methodologies and harmonized assay performance. Given the variability in ctDNA quantification, which is affected by tumor type, stage, anatomical location, tumor burden, prior treatment lines and response to therapy among others, the FDA advises manufacturers developing ctDNA-based molecular residual disease (MRD) assays for solid tumors and incorporation of ctDNA endpoints into prospective, randomized trials to support evidence generation and eventual regulatory approval (113). This lack of harmonization is a critical barrier to the broader validation and adoption of ctDNA as a decision-making tool. Therefore, ctDNA should be interpreted cautiously in early-phase trials, especially in contexts where under-detection could obscure early efficacy signals or lead to the premature termination of promising agents (118). To facilitate the clinical implementation of ctDNA as a reliable early endpoint, initiatives like the Friends of Cancer Research ctDNA for Monitoring Treatment Response (ctMoniTR) project represent important steps forward. By analyzing harmonized patient-level data from five clinical trials involving over 200 patients with advanced NSCLC treated with PD-L1 inhibitors, the ctMoniTR initiative demonstrated that on-treatment reductions in ctDNA levels were strongly associated with improved OS and PFS (119). Additionally, dynamic changes in variant allele frequency (VAF) were predictive across multiple clinical endpoints (119). These findings underscore the potential of ctDNA dynamics as an early indicator of therapeutic benefit. To advance the field, key priorities identified through such collaborative efforts include standardizing analytical methodologies and data reporting across assays, ensuring methodological transparency, systematically collecting clinicopathological data (e.g., tumor type, prior treatment), and defining minimum intervals between diagnosis and sampling (113). In conclusion, ctDNA offers a transformative, minimally invasive approach for dynamic tumor assessment in IO, with strong potential to refine patient selection and accelerate decision-making in early-phase trials. Realizing this potential critically hinges on overcoming current challenges in analytical validation and methodological standardization.

### 3.2 Serum biomarkers

Beyond ctDNA, several circulating biomarkers detectable in serum or plasma offer a minimally invasive approach to assess systemic inflammation, host immune function and tumor dynamics in patients undergoing ICI therapy. Baseline hematological parameters have been explored (120, 121). For instance, baseline absolute lymphocyte count (ALC) has been proposed as a surrogate marker of immune competence. Given the central role of lymphocytes in antitumor immunity, low pretreatment ALC may indicate impaired immune readiness and has been associated with inferior clinical outcomes in patients with NSCLC and melanoma (120, 121). Moreover, dynamic changes in lymphocyte counts following ICI initiation are complex, but their direct link to intra-tumoral immune infiltration and ICI sensitivity remains to be fully elucidated (122, 123). More consistently, derived ratios from peripheral blood counts such as the neutrophil-to-lymphocyte ratio (NLR), lymphocyte-to-monocyte ratio (LMR), and platelet-to-lymphocyte ratio (PLR), have demonstrated prognostic value across multiple tumor types (124–131). A high baseline NLR, indicative of systemic inflammation and relative lymphopenia, is consistently associated with worse outcomes with ICIs (132). Derived NLR (dNLR) has been used as an alternative to NLR and is associated with immunotherapy outcomes (133, 134). Both the dNLR and the systemic immune-inflammation index (SII), which incorporates platelet counts, have been associated with worse prognosis (132).

Markers of systemic inflammation and tumor burden, such as C-reactive protein (CRP) and lactate dehydrogenase (LDH), may also provide prognostic information. Elevated LDH at baseline often reflects increased tumor burden, tumor cell turnover, hypoxia, and anaerobic glycolysis, which contribute to an acidic and immunosuppressive TME (135). Consequently, higher LDH levels at baseline have been consistently associated with worse survival in ICI-pretreated patients (135). Interestingly, high LDH can help to identify patients with aggressive disease phenotypes less likely to benefit from immunotherapy monotherapy, guiding towards combination strategies. Similarly, elevated CRP, driven by inflammatory cytokines such as IL-6, can indicate an immunosuppressive TME, with expanded regulatory T cells (Tregs), myeloid-derived suppressor cells (MDSCs), and inhibitory cytokines (136). Elevated baseline CRP levels have been associated with adverse PFS and OS (137). An emerging phenomenon, the “CRP flare-response”, a transient rise in CRP within the first month of ICI therapy followed by a drop below baseline, has been linked to favorable outcomes, possibly indicating an effective antitumor immune activation (138). The phase 3 OAK trial, which compared atezolizumab with docetaxel in NSCLC, the CRP flare was predictive of improved survival only in the immunotherapy arm, supporting its potential as a tumor-agnostic, immunotherapy-specific marker (139). Beyond LDH and CRP, various other circulating soluble factors (e.g., sCD25, immunomodulatory cytokines, angiogenic molecules) have been linked to reduced response rates to ICIs. However, their current utility is largely exploratory due to assay variability and lack of standardization (140–147). In summary, while several serum biomarkers are readily available and inexpensive, their main limitation is the lack of specificity. Their clinical utility is often limited by a non-specific and incomplete understanding of their precise biological roles in the context of immunotherapies and multiple factors, including infections, concurrent medications, or underlying comorbidities, can influence these markers. Consequently, while they might serve as useful stratification factors or contribute to multifactorial prognostic scores, their utility for definitive patient enrichment is limited due to a high risk of misclassifying patients based on non-specific inflammatory states (148, 149).

## 4 Additional enrichment strategies

To fully optimize patient selection, molecular markers may be integrated with established, real-world clinical factors that define a patient’s overall disease state and fitness for therapy. Furthermore, clinical factors may serve as surrogates for underlying tumor immunogenicity or immune fitness, potentially improving the detection of efficacy signals. However, their application requires careful consideration of complexities and trade-offs. With increasing ICI exposure in phase 1 populations, prior immunotherapy response becomes a relevant consideration for patient selection. ICI-naïve patients generally show better outcomes, suggesting immune exhaustion or resistance in the ICI-exposed population (150). However, a history of clinical benefit from ICIs may represent a subset with intrinsically more immunogenic tumors and, subsequently, more likely to benefit from ICI rechallenge or novel immunotherapies, as demonstrated by several retrospective studies (151–153). Nevertheless, this approach risks excluding patients with primary resistance to ICIs who might respond to different combinations, especially since some novel agents (e.g., tebentafusp) may even show enhanced effects post-ICI (154–156).

The number of previous lines of therapy may also influence outcomes. Heavily pre-treated patients, common in phase 1 trials, may have a detrimentally altered TME and exhausted systemic immunity, potentially lowering the response probability to novel agents, including adoptive cell therapy (157–160). Evidence suggests earlier ICI administration often enhances efficacy (161, 162). Hence, limiting inclusion to patients with fewer prior lines might reduce heterogeneity and improve signal detection. However, this enrichment strategy raises ethical considerations regarding patient access and may limit the generalizability of early phase clinical trial results. Furthermore, the impact of treatment lines can be tumor-specific (e.g., MSS metastatic CRC showing minimal ICI benefit regardless of line) and may be outweighed by factors like performance status or overall tumor burden, emphasizing the need for integrated decision-making (163).

Baseline tumor burden and pattern of metastatic spread are also key considerations. Higher tumor burden at baseline has been associated with worse ICI outcomes (164). Specific metastatic sites, such as the liver, have been consistently associated with poorer ICI responses across multiple tumor types due to their association with a uniquely immunosuppressive TME (e.g., reduced CD8+ T-cell infiltration, increased T-cell apoptosis, systemic immune tolerance induction) (165–168). Brain metastases also present distinct challenges associated with the central nervous system (CNS) microenvironment, which is influenced by the blood-brain barrier and comprises TILs, regulatory T-cells, and glial-derived immunomodulatory cytokines, such as TGF-β (169, 170). Nevertheless, ICI combinations have shown intracranial activity in melanoma and NSCLC (169, 171–174). Inclusion of metastatic patterns as selection criteria may be balanced against the need to understand drug activity in these high-need sub-populations and would require careful integration with assessment of overall tumor burden and tumor type.

Moreover, a history of prior irAEs during prior ICI therapy presents further considerations. While irAEs during an initial ICI course have been linked to better outcomes in different retrospective studies, their predictive value for subsequent immunotherapy benefit is largely unproven (175–177). A large meta-analysis showed only weak correlation between previous irAEs and OS (178). Excluding patients with severe prior irAEs is a common safety measure, supported by recurrence rates estimated at 30% when re-treating (179).

In conclusion, clinically oriented factors like prior ICI response, treatment history, metastatic patterns, and irAEs history significantly contribute to patient heterogeneity in early-phase IO trials, complicating the interpretation of efficacy signals. Leveraging these factors for enrichment strategies requires a nuanced approach. While such strategies may offer clearer efficacy signals in selected subgroups and potentially de-risk development, they must be carefully balanced against the goals of ensuring broad patient applicability, maintaining ethical access to trials, and the critical need for prospective validation to confirm their utility in truly optimizing trial design.

## 5 Challenges and future directions

Over the past two decades, biomarker-driven clinical trials have significantly transformed oncology drug development by enabling early go/no-go decisions, real-time monitoring, and the co-development of companion diagnostics (180). Nevertheless, many current “all-comer” trial designs still fail to incorporate predictive biomarkers, resulting in potential over- or under-treatment. Addressing this limitation, while simultaneously reducing the redundancy of “me-too” drug development, will be essential to refine patient selection strategies and enhance immunotherapy efficacy across tumor types and indications, ultimately moving the field beyond its current plateau (181). Rapid advancements in NGS and multi-omics technologies have further fueled the identification of novel biomarkers, contributing to recent FDA approvals that mark a paradigm shift in precision oncology (182). As outlined in this review, these advances have driven a swift evolution in trial strategies from the foundational tissue-based markers, such as PD-L1, dMMR/MSI-H, and TMB, toward a more precise understanding of specific genomic drivers and the advent of dynamic, plasma-based monitoring. This evolution underscores a fundamental shift, where the imperative is replacing the pursuit of a single biomarker with the development of an integrated, multi-modal framework capable of capturing tumor and immune system complexity to guide the next generation of personalized treatments.

### 5.1 Challenges in trial design and biomarker validation

A central challenge remains the distinction between prognostic and predictive biomarkers. Prognostic markers provide information about clinical outcomes regardless of the therapy received, reflecting tumor or host factors that influence disease progression and survival (8, 132). Therefore, they can support clinical trial design by enabling risk stratification. In contrast, predictive biomarkers indicate the likelihood of response or resistance to a specific therapy and require formal interaction analyses to confirm the association of biomarker positivity and a differential response to a given immunotherapy versus a control regimen (183, 184). While some biomarkers may have both prognostic and predictive roles, this distinction is critical in the context of biomarker-driven clinical trial design. Concrete examples from early-phase immunotherapy trials illustrate this distinction. MSI-H/dMMR status, demonstrated in the KEYNOTE-016 phase II trial, represents a robust predictive marker of ICI benefit (57). Similarly, in the KEYNOTE-001 trial, a PD-L1 tumor proportion score (TPS) ≥50% was associated with improved ORR and PFS, supporting its role as a predictive biomarker (46). In contrast, elevated baseline LDH has consistently been a negative prognostic factor in phase I melanoma studies, reflecting tumor burden rather than treatment-specific effects (132).

As described, many widely used immunotherapy biomarkers, such as elevated baseline LDH or ctDNA levels, may function primarily as prognostic indicators or surrogates of tumor burden rather than as specific predictors of response to ICI therapy (132). This challenge is magnified in early-phase, often single-arm trials, where the absence of a control arm makes it statistically challenging to distinguish between true predictive value and prognostic influence. The implementation of narrow, biomarker-driven eligibility criteria lacking rigorous predictive validation risks over selection, which can severely limit trial enrollment and reduce the external validity of the trial´s findings to a broader, real-world patient population (185, 186). This approach carries the risk of systematically excluding subgroups of patients who might have derived benefit from treatment. Therefore, robust statistical validation, including formal interaction testing within the trial design, and a strong biological rationale are imperative before any biomarker is used for stringent patient enrichment (132, 186).

### 5.2 Operational, analytical and technological frontiers

As highlighted for established markers like PD-L1 or TMB, the path from biomarker discovery to clinical implementation is limited by significant operational barriers that further complicate the integration of novel biomarkers into early-phase immunotherapy trials. High costs, lengthy assay turnaround times, lack of harmonized testing platforms, and complex regulatory environments all contribute to delays in trial activation and limit feasibility across institutions (187). For instance, with the implementation of the new *in-vitro* diagnostic device (IVD) regulation in Europe, a 6- to 12-month delay in clinical trial activation has been estimated for clinical trial protocols using IVDs (188). These challenges are particularly pronounced for complex assays beyond standard sequencing, such as deep proteomic or spatial profiling, which face persistent hurdles related to data variability, standardization, reproducibility, and the need for robust bioinformatics infrastructure and expertise (189). Initiatives like the Clinical Proteomic Tumor Analysis Consortium (CPTAC) and transcriptomic tools such as Oncotype DX and MammaPrint have underscored the clinical value of multi-omic integration (190, 191). However, balancing the scientific advantages of biomarker-driven design with the need for broad applicability and operational efficiency remains a persistent dilemma, and the routine implementation of such approaches in early-phase trials continues to be scarce. Ultimately, clinical response to immunotherapy is rarely dictated by a single marker; instead, it emerges from the complex interplay between the tumor and the host immune system within a dynamic and heterogeneous TME. Acknowledging this complexity is the critical first step toward developing more effective biomarker strategies, compelling a move away from linear measurements. The path forward will likely require a multidimensional approach that concurrently integrates genomic data on resistance mechanisms, molecular and immune contexture profiling, and patient-specific characteristics to guide patient enrichment and support rational combination strategies in early-phase trials.

This vision is being enabled by a technological revolution that allows investigation into the tumor-immune interplay with unprecedented resolution. The most immediate evolution is in refining our genomic and transcriptomic insights. While NGS/WES provides the foundation for broad biomarkers like TMB, their application has matured to support a more specific assessment of tumor immunogenicity (192). This includes identifying specific resistance mutations, such as *STK11*, that predict ICI failure even in tumors with TMB-H, or sensitizing mutations like those in *POLE* that define a distinct “ultramutator” phenotype (124). This focus also extends to non-mutational drivers of immunogenicity; in virus-associated malignancies, for instance, the expression of viral antigens provides a source of highly potent T-cell targets. This was shown in the CheckMate 358 and CAN-2409 oncolytic virus studies, which exemplify how viral antigens can serve both as therapeutic targets and predictive tools (193, 194). To capture the functional state of this interplay, the field is moving beyond DNA alterations and toward transcriptomics (195). Pre-defined signatures offer a more nuanced predictor of a pre-existing anti-tumor immune response. For example, the 12-gene VIGex score includes 12 genes involved in immune activation and T-cell exhaustion (196). VIGex categorization has been associated with immunotherapy outcomes in patients enrolled in early phase clinical trials. Also, the 18-gene T-cell inflamed Gene Expression Profile (GEP) integrates the expression of multiple genes related to IFN-γ signaling and cytotoxic activity and has been associated with outcomes of patients treated with ICIs (197).

While deep genomic and transcriptomic profiling offers critical insights, tissue biopsies remain limited by their static, single-timepoint nature, failing to capture both spatial and temporal heterogeneity. Liquid biopsy platforms are addressing these limitations by enabling longitudinal assessment of therapeutic responses and clonal evolution. Notably, ctDNA may include data from all tumor sites, offering a systemic snapshot that can overcome spatial heterogeneity inherent in single-site tissue biopsy. This enables the non-invasive assessment of established biomarkers, such as bTMB or MSI status, and can extend beyond ctDNA to other circulating components (198). Measuring circulating exosomal PD-L1, for instance, may provide a more representative readout of the total immunosuppressive landscape than a single tissue sample (199, 200). Another challenge of single-tissue biopsy is addressing the lack of spatial context. To resolve this limitation, technologies such as multiple immunohistochemistry and immunofluorescence (mIHC/IF) and spatial transcriptomics are finally elucidating the critical architecture of the TME, distinguishing among inflamed, immune-excluded, and desert phenotypes, and revealing interactions between nearby cells (201–203). Additionally, integrating diverse biological data with advanced computational tools may enable a comprehensive vision of clinical trials, moving beyond traditional endpoints and embracing quantitative, reproducible biomarker assessment (204). Furthermore, radiomics and computational tools may also provide a non-invasive method to explore this heterogeneity on a macroscopic scale by extracting quantitative features from standard medical imaging to create a complete digital portrait of the tumor burden (205).

**Figure 1:**
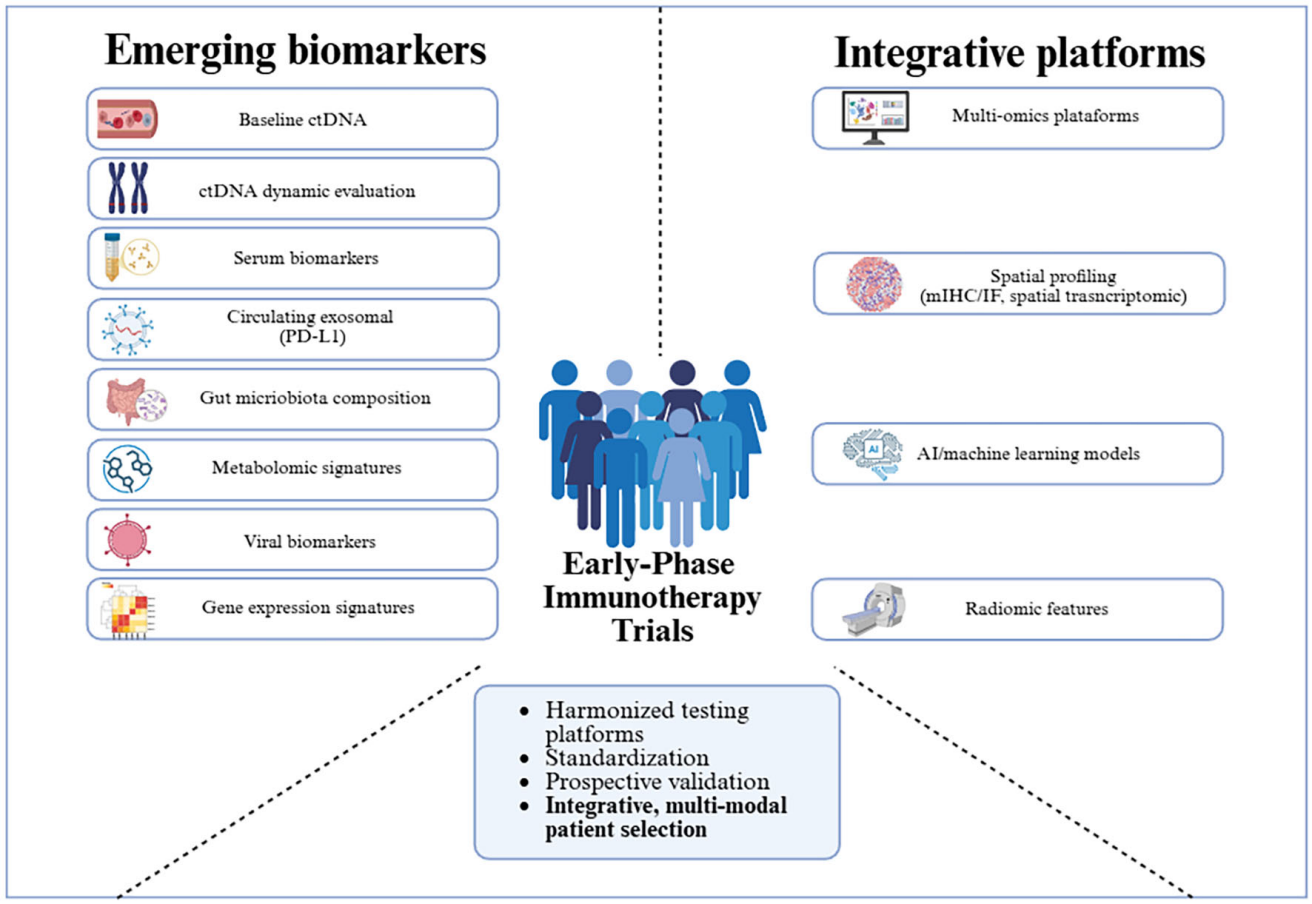
Summary of emerging biomarkers and integrative platforms under investigation in immunotherapy. To overcome the challenges associated with the implementation of novel biomarkers and multi-modal integration in early phase immunotherapy trials, efforts focused on harmonization, standardization, and prospective validation will be essential.

The biomarker landscape is further expanding beyond the tumor to encompass systemic host factors that can profoundly modulate treatment efficacy. A prominent example is the gut microbiota, where growing evidence suggests that the composition of intestinal flora can influence systemic immunity and patient response to ICIs (206, 207). For instance, taxa such as Faecalibacterium and Ruminococcus have been associated with improved outcomes. This systemic view is further enriched by metabolomics, which analyzes the metabolic products of both tumor cells and the host immune system, providing a real-time functional snapshot of the ongoing host-tumor interplay. Moreover, these discoveries not only provide information regarding potential biomarkers but may also support novel therapeutic strategies. For example, patient-derived fecal microbiota transplantation has shown promise in phase I melanoma trials (208). Artificial intelligence (AI) is emerging as necessary to integrate high-dimensional data and generate composite immune response scores. AI-driven approaches aim to overcome limitations of traditional statistical models in handling high-dimensional multi-omic data to identify predictive, not just prognostic, signatures (209, 210). For example, the proposed Predictive Biomarker Modeling Framework (PBMF) has demonstrated its capability by accurately identifying known IO biomarkers in phase 2 and 3 trials (209). Recently, SCORPIO, a machine learning model based on routine blood tests and clinical data, outperformed TMB and PD-L1 in predicting ICI benefit across multiple tumor types and settings (211). The promise of AI tools lies in their potential to uncover novel and non-linear interactions that could define responsive patient subsets for early-phase IO trials. However, the “black-box” nature of some AI algorithms, limited training datasets in early-phase settings, and the need for prospective validation limit their adoption in regulatory decision-making for early trials (212). These emerging integrative platforms, along with novel biomarkers, are summarized in **Figure 1**.

### 5.3 Regulatory challenges

Overcoming operational and regulatory hurdles is paramount to translating these multi-modal strategies from concept to clinic. The clinical maturation of less invasive biomarkers such as ctDNA, combined with the power of computational tools, already offers a pathway to faster and more efficient data generation. However, technology alone is insufficient without a concerted effort toward harmonization. Establishing unified frameworks through international collaboration is therefore essential. Landmark initiatives, such as the guidelines developed for ctDNA by Friends of Cancer Research and the FDA, provide a blueprint for standardizing data acquisition and reporting (213, 214). Such standardization is a prerequisite for streamlining regulatory processes, reducing clinical trial activation times, and building the large, high-quality datasets needed to robustly validate emerging biomarkers. In Europe, the *In Vitro* Diagnostic Regulation (IVDR) framework exemplifies this duality: while it enhances diagnostic assays’ reliability and patient safety through harmonized standards, its stringent requirements have also introduced practical delays in the activation of early-phase trials (215). Specifically, the regulation’s rigorous validation demands for laboratory-developed tests, which are essential for novel biomarkers, create significant cost and logistical hurdles that affect academic-led research. Ultimately, these collaborative frameworks between academia, industry and regulatory authorities will be key to accelerating the cycle of discovery, validation and clinical implementation. However, open questions remain on whether exploratory biomarkers for hypothesis generation require undergoing the complete clinical validation framework. In this regard, stringent criteria at early stages may hinder innovation, whereas inadequate rigor can promote non-reproducible findings. It may become essential to adopt a balanced approach that fosters innovation while maintaining methodological integrity.

## 6 Conclusion

Optimizing early-phase IO trials requires a paradigm shift, integrating genomic, transcriptomic, spatial, and systemic host data into a cohesive patient portrait, where adaptive trial designs and collaborative, transparent validation efforts converge. While this multi-dimensional approach offers exciting prospects for precision IO, translating these into validated, actionable tools for selecting patient populations in early-phase trials remains a significant challenge. Overcoming hurdles in analytical standardization, achieving robust prospective validation and ensuring their practical and economic feasibility are key to their successful clinical integration. Ultimately, focusing on these aspects is essential to move the path forward from exploratory observations into strategies that accelerate the development of next-generation immunotherapies.

## Glossary

ICIs: Immune checkpoint inhibitors
mAb: Monoclonal antibody
anti-PD-1: anti-programmed cell death 1
anti-PD-L1: anti-programmed cell death ligand 1
irAEs: Immune-related adverse events
IO: Immuno-oncology
MSI-H: Microsatellite instability-high
dMMR: Mismatch repair–deficient
FDA: U.S. Food and Drug Administration
TMB: Tumor mutational burden
TMB-H: Tumor mutational burden high
Mb: Megabase
ORR: Overall response rate
PD-L1: Programmed death-ligand 1
IHQ: Immunohistochemistry
NSCLC: Non-small cell lung cancer
TNBC: Triple-negative breast cancer
HNSCC: Head and neck squamous cell carcinoma
TCs: Tumor cells
ICs: Immune cells
TPS: Tumor Proportion Score
CPS: Combined Positive Score
RCC: Renal cell carcinoma
OS: Overall survival
MMR: Mismatch repair
EC: Endometrial cancer
CRC: Colorectal cancer
PCR: Polymerase chain reaction
NGS: Next-generation sequencing
PFS: Progression-free survival
WGS/WES: Whole-genome/exome sequencing
tTMB: Tissue-based TMB
bTMB: Blood TMB
ctDNA: Circulating tumor DNA
EDMs: Exonuclease domains
POLE: DNA polymerases epsilon
POLD1: DNA polymerases delta 1
TME: Tumor microenvironment
STK11/LKB1: Serine/Threonine kinase 11
MRD: Molecular residual disease
ctMoniTR: ctDNA for Monitoring Treatment Response
VAF: Variant allele frequency
ALC: absolute lymphocyte count
NLR: Neutrophil-to-lymphocyte ratio
LMR: Lymphocyte-to-monocyte ratio
PLR: Platelet-to-lymphocyte ratio
dNLR: Derived NLR
SII: Systemic immune-inflammation index
CRP: C-reactive protein
LDH: Lactate dehydrogenase
Tregs: Regulatory T cells
MDSCs: Myeloid-derived suppressor cells
CNS: Central nervous system
IVD: *In-vitro* diagnostic device
CPTAC: Clinical Proteomic Tumor Analysis Consortium
GEP: Gene Expression Profile
mIHC/IF: Immunohistochemistry and immunofluorescence
AI: Artificial intelligence
PBMF: Predictive Biomarker Modeling Framework

## References

[1] Hodi FS O’Day SJ McDermott DF Weber RW Sosman JA Haanen JB. Improved survival with ipilimumab in patients with metastatic melanoma. N Engl J Med. (2010) 363:711–23. doi: 10.1056/NEJMoa1003466, PMID: 20525992 PMC3549297

[2] Robert C Long GV Brady B Dutriaux C Maio M Mortier L. Nivolumab in previously untreated melanoma without BRAF mutation. N Engl J Med. (2015) 372:320–30. doi: 10.1056/NEJMoa1412082, PMID: 25399552

[3] Borghaei H Paz-Ares L Horn L Spigel DR Steins M Ready NE. Nivolumab versus Docetaxel in advanced nonsquamous non-small-cell lung cancer. N Engl J Med. (2015) 373:1627–39. doi: 10.1056/NEJMoa1507643, PMID: 26412456 PMC5705936

[4] Reck M Rodríguez-Abreu D Robinson AG Hui R Csőszi T Fülöp A. Pembrolizumab versus chemotherapy for PD-L1-positive non-small-cell lung cancer. N Engl J Med. (2016) 375:1823–33. doi: 10.1056/NEJMoa1606774, PMID: 27718847

[5] Emens LA Ascierto PA Darcy PK Demaria S Eggermont AMM Redmond WL. Cancer immunotherapy: opportunities and challenges in the rapidly evolving clinical landscape. Eur J Cancer. (2017) 81:116–29. doi: 10.1016/j.ejca.2017.01.035, PMID: 28623775

[6] Champiat S Dercle L Ammari S Massard C Hollebecque A Postel-Vinay S. Hyperprogressive disease is a new pattern of progression in cancer patients treated by anti-PD-1/PD-L1. Clin Cancer Res. (2017) 23:1920–8. doi: 10.1158/1078-0432.CCR-16-1741, PMID: 27827313

[7] Kato S Goodman A Walavalkar V Barkauskas DA Sharabi A Kurzrock R. Hyperprogressors after immunotherapy: analysis of genomic alterations associated with accelerated growth rate. Clin Cancer Res. (2017) 23:4242–50. doi: 10.1158/1078-0432.CCR-16-3133, PMID: 28351930 PMC5647162

[8] Fountzilas E Kurzrock R Vo HH Tsimberidou AM. Wedding of molecular alterations and immune checkpoint blockade: genomics as a matchmaker. J Natl Cancer Inst. (2021) 113:1634–47. doi: 10.1093/jnci/djab066, PMID: 33823006 PMC9890928

[9] Spencer KR Wang J Silk AW Ganesan S Kaufman HL Mehnert JM. Biomarkers for immunotherapy: current developments and challenges. Am Soc Clin Oncol Educ Book. (2016) 36):e493–503. doi: 10.1200/EDBK_160766, PMID: 27249758

[10] Morchón-Araujo D Catani G Mirallas O Pretelli G Sánchez-Pérez V Vieito M. Emerging immunotherapy targets in early drug development. Int J Mol Sci. (2025) 26:5394. doi: 10.3390/ijms26115394, PMID: 40508202 PMC12155519

[11] Cannarile MA Karanikas V Reis B Mancao C Lagkadinou E Rüttinger D. Facts and hopes on biomarkers for successful early clinical immunotherapy trials: innovative patient enrichment strategies. Clin Cancer Res. (2024) 30:1448–56. doi: 10.1158/1078-0432.CCR-23-1530, PMID: 38100047

[12] Wong CH Siah KW Lo AW. Estimation of clinical trial success rates and related parameters. Biostatistics. (2019) 20:273–86. doi: 10.1093/biostatistics/kxx069, PMID: 29394327 PMC6409418

[13] Sertkaya A Wong HH Jessup A Beleche T. Key cost drivers of pharmaceutical clinical trials in the United States. Clin Trials. (2016) 13:117–26. doi: 10.1177/1740774515625964, PMID: 26908540

[14] Fountzilas E Vo HH Mueller P Kurzrock R Tsimberidou AM. Correlation between biomarkers and treatment outcomes in diverse cancers: a systematic review and meta-analysis of phase I and II immunotherapy clinical trials. Eur J Cancer. (2023) 189:112927. doi: 10.1016/j.ejca.2023.05.015, PMID: 37364526 PMC10528229

[15] Marcus L Lemery SJ Keegan P Pazdur R. FDA approval summary: pembrolizumab for the treatment of microsatellite instability-high solid tumors. Clin Cancer Res. (2019) 25:3753–8. doi: 10.1158/1078-0432.CCR-18-4070, PMID: 30787022

[16] Le DT Durham JN Smith KN Wang H Bartlett BR Aulakh LK. Mismatch-repair deficiency predicts response of solid tumors to PD-1 blockade. Science. (2017) 357:409–13. doi: 10.1126/science.aan6733, PMID: 28596308 PMC5576142

[17] Goodman AM Kato S Bazhenova L Patel SP Frampton GM Miller V. Tumor mutational burden as an independent predictor of response to immunotherapy in diverse cancers. Mol Cancer Ther. (2017) 16:2598–608. doi: 10.1158/1535-7163.MCT-17-0386, PMID: 28835386 PMC5670009

[18] Legrand FA Gandara DR Mariathasan S Powles T He X Zhang W. Association of high tissue TMB and atezolizumab efficacy across multiple tumor types. JCO. (2018) 36:12000–0. doi: 10.1200/JCO.2018.36.15_suppl.12000

[19] Marcus L Fashoyin-Aje LA Donoghue M Yuan M Rodriguez L Gallagher PS. FDA approval summary: Pembrolizumab for the treatment of tumor mutational burden-high solid tumors. Clin Cancer Res. (2021) 27:4685–9. doi: 10.1158/1078-0432.CCR-21-0327, PMID: 34083238 PMC8416776

[20] Marabelle A Fakih M Lopez J Shah M Shapira-Frommer R Nakagawa K. Association of tumour mutational burden with outcomes in patients with advanced solid tumours treated with pembrolizumab: prospective biomarker analysis of the multicohort, open-label, phase 2 KEYNOTE-158 study. Lancet Oncol. (2020) 21:1353–65. doi: 10.1016/S1470-2045(20)30445-9, PMID: 32919526

[21] Health C for D and R. List of Cleared or Approved Companion Diagnostic Devices (*In Vitro* and Imaging Tools). FDA (2025). Available online at: https://www.fda.gov/medical-devices/in-vitro-diagnostics/list-cleared-or-approved-companion-diagnostic-devices-in-vitro-and-imaging-tools (Accessed April 23, 2025).

[22] Sahin IH Akce M Alese O Shaib W Lesinski GB El-Rayes B. Immune checkpoint inhibitors for the treatment of MSI-H/MMR-D colorectal cancer and a perspective on resistance mechanisms. Br J Cancer. (2019) 121:809–18. doi: 10.1038/s41416-019-0599-y, PMID: 31607751 PMC6889302

[23] Westcott PMK Muyas F Hauck H Smith OC Sacks NJ Ely ZA. Mismatch repair deficiency is not sufficient to elicit tumor immunogenicity. Nat Genet. (2023) 55:1686–95. doi: 10.1038/s41588-023-01499-4, PMID: 37709863 PMC10562252

[24] Li HD Cuevas I Zhang M Lu C Alam MM Fu YX. Polymerase-mediated ultramutagenesis in mice produces diverse cancers with high mutational load. J Clin Invest. (2018) 128:4179–91. doi: 10.1172/JCI122095, PMID: 30124468 PMC6118636

[25] Zaretsky JM Garcia-Diaz A Shin DS Escuin-Ordinas H Hugo W Hu-Lieskovan S. Mutations associated with acquired resistance to PD-1 blockade in melanoma. N Engl J Med. (2016) 375:819–29. doi: 10.1056/NEJMoa1604958, PMID: 27433843 PMC5007206

[26] Sade-Feldman M Jiao YJ Chen JH Rooney MS Barzily-Rokni M Eliane JP. Resistance to checkpoint blockade therapy through inactivation of antigen presentation. Nat Commun. (2017) 8:1136. doi: 10.1038/s41467-017-01062-w, PMID: 29070816 PMC5656607

[27] Skoulidis F Goldberg ME Greenawalt DM Hellmann MD Awad MM Gainor JF. STK11/LKB1 mutations and PD-1 inhibitor resistance in KRAS-mutant lung adenocarcinoma. Cancer Discov. (2018) 8:822–35. doi: 10.1158/2159-8290.CD-18-0099, PMID: 29773717 PMC6030433

[28] Hasegawa T Yanagitani N Ninomiya H Sakamoto H Tozuka T Yoshida H. Association between the efficacy of pembrolizumab and low STK11/LKB1 expression in high-PD-L1-expressing non-small-cell lung cancer. In Vivo. (2020) 34:2997–3003. doi: 10.21873/invivo.12131, PMID: 32871843 PMC7652526

[29] Chen X Su C Ren S Zhou C Jiang T. Pan-cancer analysis of KEAP1 mutations as biomarkers for immunotherapy outcomes. Ann Transl Med. (2020) 8:141. doi: 10.21037/atm.2019.11.52Kato, PMID: 32175433 PMC7048975

[30] Kato S Ross JS Gay L Dayyani F Roszik J Subbiah V. Analysis of MDM2 amplification: next-generation sequencing of patients with diverse Malignancies. JCO Precis Oncol. (2018) 2018 (2):1-14. doi: 10.1200/PO.17.00235, PMID: 30148248 PMC6106866

[31] Sun D Qian H Li J Xing P. Targeting MDM2 in Malignancies is a promising strategy for overcoming resistance to anticancer immunotherapy. J BioMed Sci. (2024) 31:17. doi: 10.1186/s12929-024-01004-x, PMID: 38281981 PMC10823613

[32] Peng W Chen JQ Liu C Malu S Creasy C Tetzlaff MT. Loss of PTEN promotes resistance to T cell-mediated immunotherapy. Cancer Discov. (2016) 6:202–16. doi: 10.1158/2159-8290.CD-15-0283, PMID: 26645196 PMC4744499

[33] Spranger S Bao R Gajewski TF. Melanoma-intrinsic β-catenin signalling prevents anti-tumour immunity. Nature. (2015) 523:231–5. doi: 10.1038/nature14404, PMID: 25970248

[34] Muto S Enta A Maruya Y Inomata S Yamaguchi H Mine H. Wnt/β-catenin signaling and resistance to immune checkpoint inhibitors: from non-small-cell lung cancer to other cancers. Biomedicines. (2023) 11:190. doi: 10.3390/biomedicines11010190, PMID: 36672698 PMC9855612

[35] Cardeña-Gutiérrez A López Barahona M. Predictive biomarkers of severe immune-related adverse events with immune checkpoint inhibitors: prevention, underlying causes, intensity, and consequences. Front Med (Lausanne). (2022) 9:908752. doi: 10.3389/fmed.2022.908752, PMID: 35774996 PMC9237384

[36] Spreafico A Hansen AR Abdul Razak AR Bedard PL Siu LL. The future of clinical trial design in oncology. Cancer Discov. (2021) 11:822–37. doi: 10.1158/2159-8290.CD-20-1301, PMID: 33811119 PMC8099154

[37] Kluger HM Zito CR Turcu G Baine MK Zhang H Adeniran A. PD-L1 studies across tumor types, its differential expression and predictive value in patients treated with immune checkpoint inhibitors. Clin Cancer Res. (2017) 23:4270–9. doi: 10.1158/1078-0432.CCR-16-3146, PMID: 28223273 PMC5540774

[38] Yang F Wang JF Wang Y Liu B Molina JR. Comparative analysis of predictive biomarkers for PD-1/PD-L1 inhibitors in cancers: developments and challenges. Cancers (Basel). (2021) 14:109. doi: 10.3390/cancers14010109, PMID: 35008273 PMC8750062

[39] Meng X Huang Z Teng F Xing L Yu J. Predictive biomarkers in PD-1/PD-L1 checkpoint blockade immunotherapy. Cancer Treat Rev. (2015) 41:868–76. doi: 10.1016/j.ctrv.2015.11.001, PMID: 26589760

[40] Madore J Vilain RE Menzies AM Kakavand H Wilmott JS Hyman J. PD-L1 expression in melanoma shows marked heterogeneity within and between patients: implications for anti-PD-1/PD-L1 clinical trials. Pigment Cell Melanoma Res. (2015) 28:245–53. doi: 10.1111/pcmr.12340, PMID: 25477049

[41] Di Federico A Alden SL Smithy JW Ricciuti B Alessi JV Wang X. Intrapatient variation in PD-L1 expression and tumor mutational burden and the impact on outcomes to immune checkpoint inhibitor therapy in patients with non-small-cell lung cancer. Ann Oncol. (2024) 35:902–13. doi: 10.1016/j.annonc.2024.06.014, PMID: 38950679

[42] Frank MS Bødtger U Høegholm A Stamp IM Gehl J. Re-biopsy after first line treatment in advanced NSCLC can reveal changes in PD-L1 expression. Lung Cancer. (2020) 149:23–32. doi: 10.1016/j.lungcan.2020.08.020, PMID: 32949828

[43] Li W Song P Guo L Liu X Guo C Ying J. Clinical significance of ≥ 50% PD-L1 expression with the SP263 monoclonal antibody in non-small cell lung cancer patients. Thorac Cancer. (2019) 10:175–82. doi: 10.1111/1759-7714.12929, PMID: 30536734 PMC6360219

[44] Hong L Negrao MV Dibaj SS Chen R Reuben A Bohac JM. Programmed death-ligand 1 heterogeneity and its impact on benefit from immune checkpoint inhibitors in NSCLC. J Thorac Oncol. (2020) 15:1449–59. doi: 10.1016/j.jtho.2020.04.026, PMID: 32389639

[45] Topalian SL Hodi FS Brahmer JR Gettinger SN Smith DC McDermott DF. Safety, activity, and immune correlates of anti-PD-1 antibody in cancer. N Engl J Med. (2012) 366:2443–54. doi: 10.1056/NEJMoa1200690, PMID: 22658127 PMC3544539

[46] Garon EB Rizvi NA Hui R Leighl N Balmanoukian AS Eder JP. Pembrolizumab for the treatment of non-small-cell lung cancer. N Engl J Med. (2015) 372:2018–28. doi: 10.1056/NEJMoa1501824, PMID: 25891174

[47] Herbst RS Garon EB Kim DW Cho BC Gervais R Perez-Gracia JL. Five year survival update from KEYNOTE-010: Pembrolizumab versus docetaxel for previously treated, programmed death-ligand 1-positive advanced NSCLC. J Thorac Oncol. (2021) 16:1718–32. doi: 10.1016/j.jtho.2021.05.001, PMID: 34048946

[48] Hellmann MD Paz-Ares L Bernabe Caro R Zurawski B Kim SW Carcereny Costa E. Nivolumab plus ipilimumab in advanced non-small-cell lung cancer. N Engl J Med. (2019) 381:2020–31. doi: 10.1056/NEJMoa1910231, PMID: 31562796

[49] Burtness B Harrington KJ Greil R Soulières D Tahara M de Castro G Jr. Pembrolizumab alone or with chemotherapy versus cetuximab with chemotherapy for recurrent or metastatic squamous cell carcinoma of the head and neck (KEYNOTE-048): a randomized, open-label, phase 3 study. Lancet. (2019) 394:1915–28. doi: 10.1016/S0140-6736(19)32591-7, PMID: 31679945

[50] Janjigian YY Shitara K Moehler M Garrido M Salman P Shen L. First-line nivolumab plus chemotherapy versus chemotherapy alone for advanced gastric, gastro-oesophageal junction, and oesophageal adenocarcinoma (CheckMate 649): a randomised, open-label, phase 3 trial. Lancet. (2021) 398:27–40. doi: 10.1016/S0140-6736(21)00797-2, PMID: 34102137 PMC8436782

[51] Rha SY Oh DY Yañez P Bai Y Ryu MH Lee J. KEYNOTE-859 investigators. Pembrolizumab plus chemotherapy versus placebo plus chemotherapy for HER2-negative advanced gastric cancer (KEYNOTE-859): a multicentre, randomised, double-blind, phase 3 trial. Lancet Oncol. (2023) 24:1181–95. doi: 10.1016/S1470-2045(23)00515-6, PMID: 37875143

[52] André T Shiu KK Kim TW Jensen BV Jensen LH Punt CJA. Pembrolizumab versus chemotherapy in microsatellite instability-high or mismatch repair-deficient metastatic colorectal cancer: 5-year follow-up from the randomized phase III KEYNOTE-177 study. Ann Oncol. (2025) 36:277–84. doi: 10.1016/j.annonc.2024.11.012, PMID: 39631622

[53] Andre T Elez E Van Cutsem E Jensen LH Bennouna J Mendez G. Nivolumab plus ipilimumab in microsatellite-instability-high metastatic colorectal cancer. N Engl J Med. (2024) 391:2014–26. doi: 10.1056/NEJMoa2402141, PMID: 39602630

[54] Monk BJ Colombo N Tewari KS Dubot C Caceres MV Hasegawa K. First-line pembrolizumab + Chemotherapy versus placebo + Chemotherapy for persistent, recurrent, or metastatic cervical cancer: final overall survival results of KEYNOTE-826. J Clin Oncol. (2023) 41:5505–11. doi: 10.1200/JCO.23.00914, PMID: 37910822

[55] Balar AV Castellano D O'Donnell PH Grivas P Vuky J Powles T. First-line pembrolizumab in cisplatin-ineligible patients with locally advanced and unresectable or metastatic urothelial cancer (KEYNOTE-052): a multicentre, single-arm, phase 2 study. Lancet Oncol. (2017) 18:1483–92. doi: 10.1016/S1470-2045(17)30616-2, PMID: 28967485

[56] Rosenberg JE Galsky MD Powles T Petrylak DP Bellmunt J Loriot Y. Atezolizumab monotherapy for metastatic urothelial carcinoma: final analysis from the phase II IMvigor210 trial. ESMO Open. (2024) 9:103972. doi: 10.1016/j.esmoop.2024.103972, PMID: 39642637 PMC11667038

[57] Le DT Uram JN Wang H Bartlett BR Kemberling H Eyring AD. PD-1 blockade in tumors with mismatch-repair deficiency. N Engl J Med. (2015) 372:2509–20. doi: 10.1056/NEJMoa1500596, PMID: 26028255 PMC4481136

[58] Marabelle A Le DT Ascierto PA Di Giacomo AM De Jesus-Acosta A Delord JP. Efficacy of pembrolizumab in patients with noncolorectal high microsatellite instability/mismatch repair-deficient cancer: results from the phase II KEYNOTE-158 study. J Clin Oncol. (2020) 38:1–10. doi: 10.1200/JCO.19.02105, PMID: 31682550 PMC8184060

[59] Herbst RS Soria JC Kowanetz M Fine GD Hamid O Gordon MS. Predictive correlates of response to the anti-PD-L1 antibody MPDL3280A in cancer patients. Nature. (2014) 515:563–7. doi: 10.1038/nature14011, PMID: 25428504 PMC4836193

[60] Brahmer J Reckamp KL Baas P Crinò L Eberhardt WE Poddubskaya E. Nivolumab versus docetaxel in advanced squamous-cell non–small-cell lung cancer. N Engl J Med. (2015) 373:123–35. doi: 10.1056/NEJMoa1504627, PMID: 26028407 PMC4681400

[61] Motzer RJ Escudier B George S Hammers HJ Srinivas S Tykodi SS. Nivolumab versus everolimus in patients with advanced renal cell carcinoma: Updated results with long-term follow-up of the randomized, open-label, phase 3 CheckMate 025 trial. Cancer. (2020) 126:4156–67. doi: 10.1002/cncr.33033, PMID: 32673417 PMC8415096

[62] Rossi A Pilotto S Carbognin L Ferrara MG Belluomini L Daniele G. Modern challenges for early-phase clinical trial design and biomarker discovery in metastatic non-small-cell lung cancer. J Mol Pathol. (2021) 2:207–22. doi: 10.3390/jmp2030018

[63] Hause RJ Pritchard CC Shendure J Salipante SJ. Classification and characterization of microsatellite instability across 18 cancer types. Nat Med. (2016) 22:1342–50. doi: 10.1038/nm.4191, PMID: 27694933

[64] Shimozaki K Hayashi H Tanishima S Horie S Chida A Tsugaru K. Concordance analysis of microsatellite instability status between polymerase chain reaction based testing and next generation sequencing for solid tumors. Sci Rep. (2021) 11:20003. doi: 10.1038/s41598-021-99364-z, PMID: 34625576 PMC8501090

[65] De' Angelis GL Bottarelli L Azzoni C De' Angelis N Leandro G Di Mario F. Microsatellite instability in colorectal cancer. Acta Biomed. (2018) 89:97–101. doi: 10.23750/abm.v89i9-S.7960, PMID: 30561401 PMC6502181

[66] Guyot D'Asnières De Salins A Tachon G Cohen R Karayan-Tapon L Junca A Frouin E. Discordance between immunochemistry of mismatch repair proteins and molecular testing of microsatellite instability in colorectal cancer. ESMO Open. (2021) 6:100120. doi: 10.1016/j.esmoop.2021.100120, PMID: 33930657 PMC8102173

[67] Ali-Fehmi R Krause HB Morris RT Wallbillich JJ Corey L Bandyopadhyay S. Analysis of concordance between next-generation sequencing assessment of microsatellite instability and immunohistochemistry-mismatch repair from solid tumors. JCO Precis Oncol. (2024) 8:e2300648. doi: 10.1200/PO.23.00648, PMID: 39565978 PMC11594015

[68] Roberts SA Gordenin DA. Hypermutation in human cancer genomes: footprints and mechanisms. Nat Rev Cancer. (2014) 14:786–800. doi: 10.1038/nrc3816, PMID: 25568919 PMC4280484

[69] Ma X Dong L Liu X Ou K Yang L. POLE/POLD1 mutation and tumor immunotherapy. J Exp Clin Cancer Res. (2022) 41:216. doi: 10.1186/s13046-022-02422-1, PMID: 35780178 PMC9250176

[70] FDA. Approval Timeline of Active Immunotherapies. CRI. Cancer Research Institute (2025). Available online at: https://www.cancerresearch.org/regulatory-approval-timeline-of-active-immunotherapies. (Accessed July 5, 2025) FDA.

[71] Ambrosini M Manca P Nasca V Sciortino C Ghelardi F Seligmann JF. Epidemiology, pathogenesis, biology and evolving management of MSI-H/dMMR cancers. Nat Rev Clin Oncol. (2025) 22:385–407. doi: 10.1038/s41571-025-01015-z, PMID: 40181086

[72] Budczies J Kazdal D Menzel M Beck S Kluck K Altbürger C. Tumour mutational burden: clinical utility, challenges and emerging improvements. Nat Rev Clin Oncol. (2024) 21:725–42. doi: 10.1038/s41571-024-00932-9, PMID: 39192001

[73] Sha D Jin Z Budczies J Kluck K Stenzinger A Sinicrope FA. Tumor mutational burden as a predictive biomarker in solid tumors. Cancer Discov. (2020) 10:1808–25. doi: 10.1158/2159-8290.CD-20-0522, PMID: 33139244 PMC7710563

[74] Buchhalter I Rempel E Endris V Allgäuer M Neumann O Volckmar AL. Size matters: dissecting key parameters for panel-based tumor mutational burden analysis. Int J Cancer. (2019) 144:848–58. doi: 10.1002/ijc.31878, PMID: 30238975

[75] Merino DM McShane LM Fabrizio D Funari V Chen SJ White JR. Establishing guidelines to harmonize tumor mutational burden (TMB): in silico assessment of variation in TMB quantification across diagnostic platforms: phase I of the Friends of Cancer Research TMB Harmonization Project. J Immunother Cancer. (2020) 8:e000147. doi: 10.1136/jitc-2019-000147, PMID: 32217756 PMC7174078

[76] Stenzinger A Endris V Budczies J Merkelbach-Bruse S Kazdal D Dietmaier W. Harmonization and standardization of panel-based tumor mutational burden measurement: real-world results and recommendations of the Quality in Pathology Study. J Thorac Oncol. (2020) 15:1177–89. doi: 10.1016/j.jtho.2020.01.023, PMID: 32119917

[77] Lee KW Van Cutsem E Bang YJ Fuchs CS Kudaba I Garrido M. Association of tumor mutational burden with efficacy of pembrolizumab ± chemotherapy as first-line therapy for gastric cancer in the phase III KEYNOTE-062 study. Clin Cancer Res. (2022) 28:3489–98. doi: 10.1158/1078-0432.CCR-22-012, PMID: 35657979

[78] Shitara K Özgüroğlu M Bang YJ Di Bartolomeo M Mandalà M Ryu MH. Pembrolizumab versus paclitaxel for previously treated, advanced gastric or gastro-oesophageal junction cancer (KEYNOTE-061): a randomised, open-label, controlled, phase 3 trial. Lancet. (2018) 392:123–33. doi: 10.1016/S0140-6736(18)31257-1, PMID: 29880231

[79] Zandberg DP Algazi AP Jimeno A Good JS Fayette J Bouganim N. Durvalumab for recurrent or metastatic head and neck squamous cell carcinoma: results from a single-arm, phase II study in patients with ≥25% tumour cell PDL1 expression who have progressed on platinum-based chemotherapy. Eur J Cancer. (2019) 107:142–52. doi: 10.1016/j.ejca.2018.11.015, PMID: 30576970

[80] Siu LL Even C Mesía R Remenar E Daste A Delord JP. Safety and efficacy of durvalumab with or without tremelimumab in patients with PD-L1-low/ negative recurrent or metastatic HNSCC: the phase 2 CONDOR randomized clinical trial. JAMA Oncol. (2019) 5:195–203. doi: 10.1001/jamaoncol.2018.4628, PMID: 30383184 PMC6439564

[81] Wildsmith S Li W Wu S Stewart R Morsli N Raja R. Tumor mutational burden as a predictor of survival with durvalumab and/or tremelimumab treatment in recurrent or metastatic head and neck squamous cell carcinoma. Clin Cancer Res. (2023) 29:2066–74. doi: 10.1158/1078-0432.CCR-22-2765, PMID: 36806911 PMC10233352

[82] Kawazoe A Itahashi K Yamamoto N Kotani D Kuboki Y Taniguchi H. TAS-116 (Pimitespib), an oral HSP90 inhibitor, in combination with nivolumab in patients with colorectal cancer and other solid tumors: an open-label, dose-finding, and expansion phase Ib trial (EPOC1704). Clin Cancer Res. (2021) 27:6709–15. doi: 10.1158/1078-0432.CCR-21-1929, PMID: 34593531

[83] Yarchoan M Hopkins A Jaffee EM. Tumor mutational burden and response rate to PD-1 inhibition. N Engl J Med. (2017) 377:2500–1. doi: 10.1056/NEJMc1713444, PMID: 29262275 PMC6549688

[84] McGrail DJ Pilié PG Rashid NU Voorwerk L Slagter M Kok M. High tumor mutation burden fails to predict immune checkpoint blockade response across all cancer types. Ann Oncol. (2021) 32:661–72. doi: 10.1016/j.annonc.2021.02.006, PMID: 33736924 PMC8053682

[85] Gandara DR Agarwal N Gupta S Klempner SJ Andrews MC Mahipal A. Tumor mutational burden and survival on immune checkpoint inhibition in >8000 patients across 24 cancer types. J Immunother Cancer. (2025) 13:e010311. doi: 10.1136/jitc-2024-010311, PMID: 39915003 PMC11815411

[86] Samstein RM Lee CH Shoushtari AN Hellmann MD Shen R Janjigian YY. Tumor mutational load predicts survival after immunotherapy across multiple cancer types. Nat Genet. (2019) 51:202–6. doi: 10.1038/s41588-018-0312-8, PMID: 30643254 PMC6365097

[87] Carbone DP Reck M Paz-Ares L Creelan B Horn L Steins M. First-line nivolumab in stage IV or recurrent non-small-cell lung cancer. N Engl J Med. (2017) 376:2415–26. doi: 10.1056/NEJMoa1613493, PMID: 28636851 PMC6487310

[88] Hellmann MD Ciuleanu TE Pluzanski A Lee JS Otterson GA Audigier-Valette C. Nivolumab plus ipilimumab in lung cancer with a high tumor mutational burden. N Engl J Med. (2018) 378:2093–104. doi: 10.1056/NEJMoa1801946, PMID: 29658845 PMC7193684

[89] Si H Kuziora M Quinn KJ Helman E Ye J Liu F. A blood-based assay for assessment of tumor mutational burden in first-line metastatic NSCLC treatment: Results from the MYSTIC study. Clin Cancer Res. (2021) 27:1631–40. doi: 10.1158/1078-0432.CCR-20-3771, PMID: 33355200

[90] Peters S Dziadziuszko R Morabito A Felip E Gadgeel SM Cheema P. Atezolizumab versus chemotherapy in advanced or metastatic NSCLC with high blood-based tumor mutational burden: primary analysis of BFAST cohort C randomized phase 3 trial. Nat Med. (2022) 28:1831–9. doi: 10.1038/s41591-022-01933-w, PMID: 35995953 PMC9499854

[91] Friedlaender A Nouspikel T Christinat Y Ho L McKee T Addeo A. Tissue-plasma TMB comparison and plasma TMB monitoring in patients with metastatic non-small cell lung cancer receiving immune checkpoint inhibitors. Front Oncol. (2020) 10:14. doi: 10.3389/fonc.2020.0014, PMID: 32117779 PMC7028749

[92] Wang F Zhao Q Wang YN Jin Y He MM Liu ZX. Evaluation of POLE and POLD1 mutations as biomarkers for immunotherapy outcomes across multiple cancer types. JAMA Oncol. (2019) 5:1504–6. doi: 10.1001/jamaoncol.2019.2963, PMID: 31415061 PMC6696731

[93] Hwang HS Kim D Choi J. Distinct mutational profile and immune microenvironment in microsatellite-unstable and POLE-mutated tumors. J Immunother Cancer. (2021) 9:e002797. doi: 10.1136/jitc-2021-002797, PMID: 34607897 PMC8491424

[94] Jin Y Huang RJ Guan WL Wang ZQ Mai ZJ Li YH. A phase II clinical trial of toripalimab in advanced solid tumors with polymerase epsilon/polymerase delta (POLE/POLD1) mutation. Signal Transduct Target Ther. (2024) 9:227. doi: 10.1038/s41392-024-01939-5, PMID: 39218995 PMC11366758

[95] Ott PA Bang YJ Berton-Rigaud D Elez E Pishvaian MJ Rugo HS. Safety and antitumor activity of pembrolizumab in advanced programmed death ligand 1-positive endometrial cancer: results from the KEYNOTE-028 study. J Clin Oncol. (2017) 35:2535–41. doi: 10.1200/JCO.2017.72.5952, PMID: 28489510

[96] Federation Francophone de Cancerologie Digestive. Multicenter prospective cohort of tumors with POLE/POLD1 mutation(2024). Available online at: https://clinicaltrials.gov/study/NCT05103969 (Accessed May 12, 2025).

[97] A single-arm, open, multicenter phase II study of chemotherapy-sequential tislelizumab adjuvant therapy after radical resection in patients with gastric or colorectal adenocarcinoma with dMMR/MSI-H or POLE/POLD1 mutations(2023). Available online at: https://clinicaltrials.gov/study/NCT06118658 (Accessed May 12, 2025).

[98] A phase II open label study of toripalimab, a PD-1 antibody, in participants with POLE or POLD-1 mutated and non-MSI-H advanced solid tumors(2023). Available online at: https://clinicaltrials.gov/study/NCT03810339 (Accessed May 12, 2025).

[99] Wu JN Roberts CW. ARID1A mutations in cancer: another epigenetic tumor suppressor? Cancer Discov. (2013) 3:35–43. doi: 10.1158/2159-8290.CD-12-0361, PMID: 23208470 PMC3546152

[100] Jelinic P Ricca J Van Oudenhove E Olvera N Merghoub T Levine DA. Immune-active microenvironment in small cell carcinoma of the ovary, hypercalcemic type: rationale for immune checkpoint blockade. J Natl Cancer Inst. (2018) 110:787–90. doi: 10.1093/jnci/djx277, PMID: 29365144 PMC6037122

[101] Bakouny Z Braun DA Shukla SA Pan W Gao X Hou Y. Integrative molecular characterization of sarcomatoid and rhabdoid renal cell carcinoma. Nat Commun. (2021) 12:808. doi: 10.1038/s41467-021-21068-9, PMID: 33547292 PMC7865061

[102] Spiliopoulou P Yang SYC Bruce JP Wang BX Berman HK Pugh TJ. All is not lost: learning from 9p21 loss in cancer. Trends Immunol. (2022) 43:379–90. doi: 10.1016/j.it.2022.03.003, PMID: 35379580

[103] Horn S Leonardelli S Sucker A SChadendorf D Griewank KG Paschen A. Tumor CDKN2A-associated JAK2 loss and susceptibility to immunotherapy resistance. J Natl Cancer Inst. (2018) 110:677–81. doi: 10.1093/jnci/djx271, PMID: 29917141

[104] Han G Yang G Hao D Lu Y Thein K Simpson BS. 9p21 loss confers a cold tumor immune microenvironment and primary resistance to immune checkpoint therapy. Nat Commun. (2021) 12:5606. doi: 10.1038/s41467-021-25894-9, PMID: 34556668 PMC8460828

[105] Davoli T Uno H Wooten EC Elledge SJ. Tumor aneuploidy correlates with markers of immune evasion and with reduced response to immunotherapy. Science. (2017) 355:eaaf8399. doi: 10.1126/science.aaf8399, PMID: 28104840 PMC5592794

[106] Roh W Chen PL Reuben A Spencer CN Prieto PA Miller JP. Integrated molecular analysis of tumor biopsies on sequential CTLA-4 and PD-1 blockade reveals markers of response and resistance. Sci Transl Med. (2017) 9:eaah3560. doi: 10.1126/scitranslmed.aah3560, PMID: 28251903 PMC5819607

[107] Zhang C Li D Xiao B Zhou C Jiang W Tang J. B2M and JAK1/2-mutated MSI-H colorectal carcinomas can benefit from anti-PD-1 therapy. J Immunother. (2022) 45:187–93. doi: 10.1097/CJI.0000000000000417, PMID: 35343934 PMC8986629

[108] Khagi Y Goodman AM Daniels GA Patel SP Sacco AG Randall JM. Hypermutated circulating tumor DNA: correlation with response to checkpoint inhibitor-based immunotherapy. Clin Cancer Res. (2017) 23:5729–36. doi: 10.1158/1078-0432.CCR-17-1439, PMID: 28972084 PMC5678984

[109] Heitzer E Ulz P Geigl JB. Circulating tumor DNA as a liquid biopsy for cancer. Clin Chem. (2015) 61:112–23. doi: 10.1373/clinchem.2014.222679, PMID: 25388429

[110] Lee JH Long GV Boyd S Lo S Menzies AM Tembe V. Circulating tumour DNA predicts response to anti-PD1 antibodies in metastatic melanoma. Ann Oncol. (2017) 28:1130–6. doi: 10.1093/annonc/mdx026, PMID: 28327969

[111] Bratman SV Yang SYC Iafolla MAJ Liu Z Hansen AR Bedard PL. Personalized circulating tumor DNA analysis as a predictive biomarker in solid tumor patients treated with pembrolizumab. Nat Cancer. (2020) 1:873–81. doi: 10.1038/s43018-020-0096-5, PMID: 35121950

[112] De Almeida Toledo R Calahorro García AM Mirallas O Moreno A Galvao V Alonso G. Prognostic and predictive value of ultrasensitive ctDNA monitoring in a metastatic pan-cancer cohort treated with immune checkpoint inhibitors in the context of phase 1 clinical trials [Abstract. JCO. (2024) 42:2510–0. doi: 10.1200/JCO.2024.42.16_suppl.2510

[113] U.S. Food and Drug Administration. Use of circulating tumor deoxyribonucleic acid for early-stage solid tumor drug development: guidance for industry(2024). Available online at: https://www.fda.gov/regulatory-information/search-fda-guidance-documents/use-circulating-tumor-deoxyribonucleic-acid-early-stage-solid-tumor-drug-development-guidance (Accessed May 18, 2025).

[114] Kasi PM Fehringer G Taniguchi H Starling N Nakamura Y Kotani D. Impact of circulating tumor DNA–based detection of molecular residual disease on the conduct and design of clinical trials for solid tumors. JCO Precis Oncol. (2022) 6):e2100181. doi: 10.1200/PO.21.00181, PMID: 35263168 PMC8926064

[115] Rothwell DG Ayub M Cook N Thistlethwaite F Carter L Dean E. Utility of ctDNA to support patient selection for early phase clinical trials: the TARGET study. Nat Med. (2019) 25:738–43. doi: 10.1038/s41591-019-0380-z, PMID: 31011204

[116] Willis J Lefterova MI Artyomenko A Kasi PM Nakamura Y Mody K. Validation of microsatellite instability detection using a comprehensive plasma-based genotyping panel. Clin Cancer Res. (2019) 25:7035–45. doi: 10.1158/1078-0432.CCR-19-1324, PMID: 31383735

[117] Vellanki PJ Ghosh S Pathak A Fusco MJ Bloomquist EW Tang S. Regulatory implications of ctDNA in immuno-oncology for solid tumors. J ImmunoTher Cancer. (2023) 11:e005344. doi: 10.1136/jitc-2022-005344, PMID: 36796877 PMC9936292

[118] Guigal-Stephan N Lockhart B Moser T Heitzer E. A perspective review on the systematic implementation of ctDNA in phase I clinical trial drug development. J Exp Clin Cancer Res. (2025) 44:79. doi: 10.1186/s13046-025-03328-4, PMID: 40022112 PMC11871688

[119] Vega DM Nishimura KK Zariffa N Thompson JC Hoering A Cilento V. Changes in circulating tumor DNA reflect clinical benefit across multiple studies of patients with non-small-cell lung cancer treated with immune checkpoint inhibitors. JCO Precis Oncol. (2022) 6:e2100372. doi: 10.1200/PO.21.00372, PMID: 35952319 PMC9384957

[120] Li S Zhang C Pang G Wang P. Emerging blood-based biomarkers for predicting response to checkpoint immunotherapy in non-small-cell lung cancer. Front Immunol. (2020) 11:603157. doi: 10.3389/fimmu.2020.603157, PMID: 33178229 PMC7596386

[121] Huang AC Postow MA Orlowski RJ Mick R Bengsch B Manne S. T-cell invigoration to tumour burden ratio associated with anti-PD-1 response. Nature. (2017) 545:60–5. doi: 10.1038/nature22079, PMID: 28397821 PMC5554367

[122] Relecom A Merhi M Inchakalody V Uddin S Rinchai D Bedognetti D. Emerging dynamics pathways of response and resistance to PD-1 and CTLA-4 blockade: tackling uncertainty by confronting complexity. J Exp Clin Cancer Res. (2021) 40:74. doi: 10.1186/s13046-021-01872-3, PMID: 33602280 PMC7893879

[123] Valpione S Galvani E Tweedy J Mundra PA Banyard A Middlehurst P. Immune-awakening revealed by peripheral T cell dynamics after one cycle of immunotherapy. Nat Cancer. (2020) 1:210–21. doi: 10.1038/s43018-019-0022-x, PMID: 32110781 PMC7046489

[124] Mihaila RI Gheorghe AS Zob DL Stanculeanu DL. The importance of predictive biomarkers and their correlation with the response to immunotherapy in solid tumors—Impact on clinical practice. Biomedicines. (2024) 12:2146. doi: 10.3390/biomedicines12092146, PMID: 39335659 PMC11429372

[125] Ferrucci PF Gandini S Battaglia A Alfieri S Di Giacomo AM Giannarelli D. Baseline neutrophil-to-lymphocyte ratio is associated with outcome of ipilimumab-treated metastatic melanoma patients. Br J Cancer. (2015) 112:1904–10. doi: 10.1038/bjc.2015.180, PMID: 26010413 PMC4580390

[126] Diem S Schmid S Krapf M Flatz L Born D Jochum W. Neutrophil-to-Lymphocyte ratio (NLR) and Platelet-to-Lymphocyte ratio (PLR) as prognostic markers in patients with non-small cell lung cancer (NSCLC) treated with nivolumab. Lung Cancer. (2017) 111:176–81. doi: 10.1016/j.lungcan.2017.07.024, PMID: 28838390

[127] de Nonneville A Barbolosi D Andriantsoa M El-Cheikh R Duffaud F Bertucci F. Validation of neutrophil count as an algorithm-based predictive factor of progression-free survival in patients with metastatic soft tissue sarcomas treated with trabectedin. Cancers (Basel). (2019) 11:432. doi: 10.3390/cancers11030432, PMID: 30917620 PMC6468511

[128] Xiong Q Huang Z Xin L Qin B Zhao X Zhang J. Post-treatment neutrophil-to-lymphocyte ratio (NLR) predicts response to anti-PD-1/PD-L1 antibody in SCLC patients at early phase. Cancer Immunol Immunother. (2021) 70:713–20. doi: 10.1007/s00262-020-02706-5, PMID: 32910245 PMC10991352

[129] Ota Y Takahari D Suzuki T Osumi H Nakayama I Oki A. Changes in the neutrophil-to-lymphocyte ratio during nivolumab monotherapy are associated with gastric cancer survival. Cancer Chemother Pharmacol. (2020) 85:265–72. doi: 10.1007/s00280-019-04023-w, PMID: 31907646

[130] Parosanu AI Pirlog CF Slavu CO Stanciu IM Cotan H-T Vrabie RC. The prognostic value of neutrophil-to-lymphocyte ratio in patients with metastatic renal cell carcinoma. Curr Oncol. (2023) 30:2457–64. doi: 10.3390/curroncol30020187, PMID: 36826148 PMC9955537

[131] Shang J Han X Zha H Tao H Li X Yuan F. Systemic immune-inflammation index and changes of neutrophil-lymphocyte ratio as prognostic biomarkers for patients with pancreatic cancer treated with immune checkpoint blockade. Front Oncol. (2021) 11:585271. doi: 10.3389/fonc.2021.585271, PMID: 33718140 PMC7943876

[132] Cottrell TR Lotze MT Ali A Bifulco CB Capitini CM Chow LQM. Society for Immunotherapy of Cancer (SITC) consensus statement on essential biomarkers for immunotherapy clinical protocols. J Immunother Cancer. (2025) 13:e010928. doi: 10.1136/jitc-2024-010928, PMID: 40054999 PMC11891540

[133] Hernando-Calvo A Mirallas O Marmolejo D Saavedra O Vieito M Assaf Pastrana JD. Nutritional status associates with immunotherapy clinical outcomes in recurrent or metastatic head and neck squamous cell carcinoma patients. Oral Oncol. (2023) 140:106364. doi: 10.1016/j.oraloncology.2023.106364, PMID: 36989964

[134] Hernando-Calvo A García-Alvarez A Villacampa G Ortiz C Bodet D García-Patos V. Dynamics of clinical biomarkers as predictors of immunotherapy benefit in metastatic melanoma patients. Clin Transl Oncol. (2021) 23:311–7. doi: 10.1007/s12094-020-02420-9, PMID: 32562197

[135] Petrelli F Cabiddu M Coinu A Borgonovo K Ghilardi M Lonati V. Prognostic role of lactate dehydrogenase in solid tumors: a systematic review and meta-analysis of 76 studies. Acta Oncol. (2015) 54:961–70. doi: 10.3109/0284186X.2015.1043026, PMID: 25984930

[136] Iivanainen S Ahvonen J Knuuttila A Tiainen S Koivunen JP. Elevated CRP levels indicate poor progression-free and overall survival on cancer patients treated with PD-1 inhibitors. ESMO Open. (2019) 4:e000531. doi: 10.1136/esmoopen-2019-000531, PMID: 31555483 PMC6735669

[137] Fujiwara Y D’Ovidio TJ Baldwin E Joshi H Doroshow DB Galsky MD. C-reactive protein as a response biomarker to immune checkpoint blockade: A meta-analysis. J Clin Oncol. (2023) 41:2559–9. doi: 10.1200/JCO.2023.41.16_suppl.2559

[138] Barth DA Moik F Steinlechner S Posch F Mayer MC Sandner AM. Early kinetics of C reactive protein for cancer-agnostic prediction of therapy response and mortality in patients treated with immune checkpoint inhibitors: a multicenter cohort study. J Immunother Cancer. (2023) 11:e007765. doi: 10.1136/jitc-2023-007765, PMID: 38097343 PMC10729183

[139] Saal J Bald T Eckstein M Ritter M Brossart P Ellinger J. Early C-reactive protein kinetics predicts immunotherapy response in non-small cell lung cancer in the phase III OAK trial. JNCI Cancer Spectr. (2023) 7:pkad027. doi: 10.1093/jncics/pkad027, PMID: 37004206 PMC10121335

[140] Hannani D Vétizou M Enot D Rusakiewicz S Chaput N Klatzmann D. Anticancer immunotherapy by CTLA-4 blockade: obligatory contribution of IL-2 receptors and negative prognostic impact of soluble CD25. Cell Res. (2015) 25:208–24. doi: 10.1038/cr.2015.3, PMID: 25582080 PMC4650573

[141] Lee H Pal SK Reckamp K Figlin RA Yu H. STAT3: a target to enhance antitumor immune response. Curr Top Microbiol Immunol. (2011) 344:41–59. doi: 10.1007/82_2010_51, PMID: 20517723 PMC3244828

[142] Lippitz BE Harris RA. Cytokine patterns in cancer patients: A review of the correlation between interleukin 6 and prognosis. Oncoimmunology. (2016) 5:e1093722. doi: 10.1080/2162402X.2015.1093722, PMID: 27467926 PMC4910721

[143] Kang DH Park CK Chung C Oh IJ Kim YC Park D. Baseline serum interleukin-6 levels predict the response of patients with advanced non-small cell lung cancer to PD-1/PD-L1 inhibitors. Immune Netw. (2020) 20:e27. doi: 10.4110/in.2020.20.e27, PMID: 32655975 PMC7327149

[144] Wang M Zhai X Li J Guan J Xu S Li Y. The role of cytokines in predicting the response and adverse events related to immune checkpoint inhibitors. Front Immunol. (2021) 12:670391. doi: 10.3389/fimmu.2021.670391, PMID: 34367136 PMC8339552

[145] Fousek K Horn LA Palena C. Interleukin-8: A chemokine at the intersection of cancer plasticity, angiogenesis, and immune suppression. Pharmacol Ther. (2021) 219:107692. doi: 10.1016/j.pharmthera.2020.107692, PMID: 32980444 PMC8344087

[146] Wu X Giobbie-Hurder A Liao X Connelly C Connolly EM Li J. Angiopoietin-2 as a biomarker and target for immune checkpoint therapy. Cancer Immunol Res. (2017) 5:17–28. doi: 10.1158/2326-6066.CIR-16-0206, PMID: 28003187 PMC5215959

[147] Yuan J Zhou J Dong Z Tandon S Kuk D Panageas KS. Pretreatment serum VEGF is associated with clinical response and overall survival in advanced melanoma patients treated with ipilimumab. Cancer Immunol Res. (2014) 2:127–32. doi: 10.1158/2326-6066.CIR-13-0163, PMID: 24778276 PMC3991109

[148] Ashok Kumar P Basnet A. The role of laboratory tests as a prognostic marker for immune-checkpoint therapy in non-small cell lung cancer. Transl Lung Cancer Res. (2023) 12:1838–41. doi: 10.21037/tlcr-23-566, PMID: 37854158 PMC10579829

[149] Kaczmarek F Marcinkowska-Gapińska A Bartkowiak-Wieczorek J Nowak M Kmiecik M Brzezińska K. Blood-based biomarkers as predictive and prognostic factors in immunotherapy-treated patients with solid tumors—currents and perspectives. Cancers (Basel). (2025) 17:2001. doi: 10.3390/cancers17122001, PMID: 40563651 PMC12190272

[150] Martini DJ Liu Y Shabto JM Lewis C Kline MR Collins H. Clinical outcomes of advanced stage cancer patients treated with sequential immunotherapy in phase 1 clinical trials. Invest New Drugs. (2019) 37:1198–206. doi: 10.1007/s10637-019-00736-0, PMID: 30725388 PMC6684862

[151] Shreders A Joseph R Peng C Ye F Zhao S Puzanov I. Prolonged benefit from ipilimumab correlates with improved outcomes from subsequent pembrolizumab. Cancer Immunol Res. (2016) 4:569–73. doi: 10.1158/2326-6066.CIR-15-0281, PMID: 27197063 PMC4940026

[152] Perdyan A Sobocki BK Balihodzic A Dąbrowska A Kacperczyk J Rutkowski J. The effectiveness of cancer immune checkpoint inhibitor retreatment and rechallenge—a systematic review. Cancers (Basel). (2023) 15:3490. doi: 10.3390/cancers15133490, PMID: 37444600 PMC10340255

[153] Cao J Ding X Ji J Zhang L Luo C. Efficacy and safety of immune checkpoint inhibitors rechallenge in advanced solid tumors: a systematic review and meta-analysis. Front Oncol. (2024) 14:1475502. doi: 10.3389/fonc.2024.1475502, PMID: 39726701 PMC11669585

[154] Olson DJ Eroglu Z Brockstein B Poklepovic AS Bajaj M Babu S. Pembrolizumab plus ipilimumab following anti-PD-1/L1 failure in melanoma. J Clin Oncol. (2021) 39:2647–55. doi: 10.1200/JCO.21.00079, PMID: 33945288 PMC8376314

[155] VanderWalde A Bellasea SL Kendra KL Khushalani NI Campbell KM Scumpia PO. Ipilimumab with or without nivolumab in PD-1 or PD-L1 blockade refractory metastatic melanoma: a randomized phase 2 trial. Nat Med. (2023) 29:2278–85. doi: 10.1038/s41591-023-02498-y, PMID: 37592104 PMC10708907

[156] Dimitriou F Orloff MM Koch Hein EC Cheng PF Hughes IF Simeone E. Treatment sequence with tebentafusp and immune checkpoint inhibitors in patients with metastatic uveal melanoma and metastatic GNA11/GNAQ mutant melanocytic tumors. Eur J Cancer. (2025) 214:115161. doi: 10.1016/j.ejca.2024.115161, PMID: 39647344

[157] Bari S Matejcic M Kim RD Xie H Sahin IH Powers BD. Practice patterns and survival outcomes of immunotherapy for metastatic colorectal cancer. JAMA Netw Open. (2025) 8:e251186. doi: 10.1001/jamanetworkopen.2025.1186, PMID: 40111368 PMC11926646

[158] Danesi V Massa I Foca F Delmonte A Crinò L Bronte G. Real-world outcomes and treatments patterns prior and after the introduction of first-line immunotherapy for the treatment of metastatic non-small cell lung cancer. Cancers (Basel). (2022) 14:4481. doi: 10.3390/cancers14184481, PMID: 36139641 PMC9497168

[159] Long GV Atkinson V Lo SN Guminski AD Sandhu SK Brown MP. Ipilimumab plus nivolumab versus nivolumab alone in patients with melanoma brain metastases (ABC): 7-year follow-up of a multicentre, open-label, randomised, phase 2 study. Lancet Oncol. (2025) 26:320–30. doi: 10.1016/S1470-2045(24)00735-6, PMID: 39978375

[160] Seitter SJ Sherry RM Yang JC Robbins PF Shindorf ML Copeland AR. Impact of prior treatment on the efficacy of adoptive transfer of tumor-infiltrating lymphocytes in patients with metastatic melanoma. Clin Cancer Res. (2021) 27:5289–98. doi: 10.1158/1078-0432.CCR-21-1171, PMID: 34413159 PMC8857302

[161] Soto JJ Llop-Serna S Stradella A Villanueva Vázquez R Calvo M Jove M. Clinical outcomes in phase 1 clinical trials (Ph1-CT) when treating patients (pts) with novel immunotherapy (IT) agents at early lines for advanced disease. J Clin Oncol. (2023) 41:2586. doi: 10.1200/JCO.2023.41.16_suppl.2586

[162] Losurdo A Dipasquale A Giordano L Persico P Lorenzi E Di Muzio A. Refining patient selection for next-generation immunotherapeutic early-phase clinical trials with a novel and externally validated prognostic nomogram. Front Immunol. (2024) 15:1323151. doi: 10.3389/fimmu.2024.1323151, PMID: 38298193 PMC10828843

[163] Topalian SL Hodi FS Brahmer JR Gettinger SN Smith DC McDermott DF. Five-year survival and correlates among patients with advanced melanoma, renal cell carcinoma, or non-small cell lung cancer treated with nivolumab. JAMA Oncol. (2019) 5:1411–20. doi: 10.1001/jamaoncol.2019.2187, PMID: 31343665 PMC6659167

[164] Tarantino P Marra A Gandini S Minotti M Pricolo P Signorelli G. Association between baseline tumour burden and outcome in patients with cancer treated with next-generation immunoncology agents. Eur J Cancer. (2020) 139:92–8. doi: 10.1016/j.ejca.2020.08.026, PMID: 32979647

[165] Wang C Sandhu J Ouyang C Ye J Lee PP Fakih M. Clinical response to immunotherapy targeting programmed cell death receptor 1/programmed cell death ligand 1 in patients with treatment-resistant microsatellite stable colorectal cancer with and without liver metastases. JAMA Netw Open. (2021) 4:e2118416. doi: 10.1001/jamanetworkopen.2021.18416, PMID: 34369992 PMC8353537

[166] Bardakçi M Ergun Y. Immunotherapy in gastric cancer with liver metastasis: challenges and opportunities. World J Gastrointest Surg. (2024) 16:1513–6. doi: 10.4240/wjgs.v16.i6.1513, PMID: 38983315 PMC11230037

[167] Bullock AJ Schlechter BL Fakih MG Tsimberidou AM Grossman JE Gordon MS. Botensilimab plus balstilimab in relapsed/refractory microsatellite stable metastatic colorectal cancer: a phase 1 trial. Nat Med. (2024) 30:2558–67. doi: 10.1038/s41591-024-03083-7, PMID: 38871975 PMC11405281

[168] Yu J Green MD Li S Sun Y Journey SN Choi JE. Liver metastasis restrains immunotherapy efficacy *via* macrophage-mediated T cell elimination. Nat Med. (2021) 27:152–64. doi: 10.1038/s41591-020-1131-x, PMID: 33398162 PMC8095049

[169] Patel L Kolundzic N Abedalthagafi M. Progress in personalized immunotherapy for patients with brain metastasis. NPJ Precis Oncol. (2025) 9:31. doi: 10.1038/s41698-025-00812-0, PMID: 39880875 PMC11779815

[170] Schulz M Salamero-Boix A Niesel K Alekseeva T Sevenich L. Microenvironmental regulation of tumor progression and therapeutic response in brain metastasis. Front Immunol. (2019) 10:1713. doi: 10.3389/fimmu.2019.01713, PMID: 31396225 PMC6667643

[171] Tawbi HA Forsyth PA Algazi A Hamid O Hodi FS Moschos SJ. Combined nivolumab and ipilimumab in melanoma metastatic to the brain. N Engl J Med. (2018) 379:722–30. doi: 10.1056/NEJMoa1805453, PMID: 30134131 PMC8011001

[172] Reck M Ciuleanu TE Lee JS Schenker M Zurawski B Kim SW. Systemic and intracranial outcomes with first-line nivolumab plus ipilimumab in patients with metastatic NSCLC and baseline brain metastases from CheckMate 227 Part 1. J Thorac Oncol. (2023) 18:1055–69. doi: 10.1016/j.jtho.2023.04.021, PMID: 37146754

[173] Karimi E Yu MW Maritan SM Perus LJM Rezanejad M Sorin M. Single-cell spatial immune landscapes of primary and metastatic brain tumours. Nature. (2023) 614:555–63. doi: 10.1038/s41586-022-05680-3, PMID: 36725935 PMC9931580

[174] Wischnewski V Maas RR Aruffo PG Soukup K Galletti G Kornete M. Phenotypic diversity of T cells in human primary and metastatic brain tumors revealed by multiomic interrogation. Nat Cancer. (2023) 4:908–24. doi: 10.1038/s43018-023-00566-3, PMID: 37217652 PMC10293012

[175] Liang Y Xu H Liu F Li L Lin C Zhang Y. Immune-related adverse events and their effects on survival outcomes in patients with non-small cell lung cancer treated with immune checkpoint inhibitors: a systematic review and meta-analysis. Front Oncol. (2024) 14:1281645. doi: 10.3389/fonc.2024.1281645, PMID: 38887231 PMC11180722

[176] Weber JS Hodi FS Wolchok JD Topalian SL SChadendorf D Larkin J. Safety profile of nivolumab monotherapy: a pooled analysis of patients with advanced melanoma. J Clin Oncol. (2017) 35:785–92. doi: 10.1200/JCO.2015.66.1389, PMID: 28068177

[177] Zhou X Yao Z Yang H Liang N Zhang X Zhang F. Are immune-related adverse events associated with the efficacy of immune checkpoint inhibitors in patients with cancer? A systematic review and meta-analysis. BMC Med. (2020) 18:87. doi: 10.1186/s12916-020-01549-2, PMID: 32306958 PMC7169020

[178] Amoroso V Gallo F Alberti A Paloschi D Ferrari Bravo W Esposito A. Immune-related adverse events as potential surrogates of immune checkpoint inhibitors' efficacy: a systematic review and meta-analysis of randomized studies. ESMO Open. (2023) 8:100787. doi: 10.1016/j.esmoop.2023.100787, PMID: 36842300 PMC9984799

[179] Dolladille C Ederhy S Sassier M Cautela J Thuny F Cohen AA. Immune checkpoint inhibitor rechallenge after immune-related adverse events in patients with cancer. JAMA Oncol. (2020) 6:865–71. doi: 10.1001/jamaoncol.2020.0726, PMID: 32297899 PMC7163782

[180] Hodgson DR Whittaker RD Herath A Amakye D Clack G. Biomarkers in oncology drug development. Mol Oncol. (2009) 3:24–32. doi: 10.1016/j.molonc.2008.12.002, PMID: 19383364 PMC5527864

[181] Salomone F Nuccio A Ferrara R. PD-L1-overexpressing NSCLC: overcoming all-comer approach and network effect to weather the "Winter" of cancer immunotherapy. J Thorac Oncol. (2025) 20:834–8. doi: 10.1016/j.jtho.2025.04.012, PMID: 40617666

[182] Committee on the Review of Omics-Based Tests for Predicting Patient Outcomes in Clinical Trials Board on Health Care Services Board on Health Sciences Policy Institute of Medicine. Evolution of Translational Omics: Lessons Learned and the Path Forward. Micheel CM Nass SJ Omenn GS, editors. Washington (DC: National Academies Press (US (2012). doi: 10.17226/13297, PMID: 24872966

[183] Halabi S Li C Luo S. Developing and validating risk assessment models of clinical outcomes in modern oncology. JCO Precis Oncol. (2019) 3(3):1-12. doi: 10.1200/PO.19.00068, PMID: 31840130 PMC6908945

[184] Halabi S Niedzwiecki D. Advancing precision oncology through biomarker-driven trials: theory vs. practice. Chance. (2019) 32:23–31. doi: 10.1080/09332480.2019.1695438 PMC13082831 41993138

[185] Mandrekar SJ Sargent DJ. Predictive biomarker validation in practice: lessons from real trials. Clin Trials. (2010) 7:567–73. doi: 10.1177/1740774510368574, PMID: 20392785 PMC3913192

[186] Ou FS Michiels S Shyr Y Adjei AA Oberg AL. Biomarker discovery and validation: statistical considerations. J Thorac Oncol. (2021) 16:537–45. doi: 10.1016/j.jtho.2021.01.161, PMID: 33545385 PMC8012218

[187] Andre F McShane LM Michiels S Ransohoff DF Altman DG Reis-Filho JS. Biomarker studies: a call for a comprehensive biomarker study registry. Nat Rev Clin Oncol. (2011) 8:171–6. doi: 10.1038/nrclinonc.2011.4, PMID: 21364690

[188] European Federation of Pharmaceutical Industries and Associations (EFPIA). Critical impacts of IVDR implementation on patient access to clinical trials (2022). Available online at: https://efpia.eu/media/677143/efpia_ivdr-survey-slides.pdf (Accessed July 11, 2025).

[189] Piñero J Rodriguez Fraga PS Valls-Margarit J Ronzano F Accuosto P Lambea Jane R. Genomic and proteomic biomarker landscape in clinical trials. Comput Struct Biotechnol J. (2023) 21:2110–8. doi: 10.1016/j.csbj.2023.03.014, PMID: 36968019 PMC10036891

[190] Paik S Shak S Tang G Kim C Baker J Cronin M. A multigene assay to predict recurrence of tamoxifen-treated, node-negative breast cancer. N Engl J Med. (2004) 351:2817–26. doi: 10.1056/NEJMoa041588, PMID: 15591335

[191] Zhang H Liu T Zhang Z Payne SH Zhang B McDermott JE. Integrated proteogenomic characterization of human high-grade serous ovarian cancer. Cell. (2016) 166:755–65. doi: 10.1016/j.cell.2016.05.069, PMID: 27372738 PMC4967013

[192] Lin X Zong C Zhang Z Fang W Xu P. Progresses in biomarkers for cancer immunotherapy. MedComm (2020). (2023) 4:e387. doi: 10.1002/mco2.387, PMID: 37799808 PMC10547938

[193] Naumann RW Hollebecque A Meyer T Devlin MJ Oaknin A Kerger J. Safety and efficacy of nivolumab monotherapy in recurrent or metastatic cervical, vaginal, or vulvar carcinoma: results from the phase I/II checkMate 358 trial. J Clin Oncol. (2019) 37:2825–34. doi: 10.1200/JCO.19.00739, PMID: 31487218 PMC6823884

[194] Wen P Duault C Gonzalez-Kozlova E Brennan K Holmes T Kim-Schulze S. 1477 First efficacy and multi-omic analysis data from phase 1 clinical trial of oncolytic viral immunotherapy with CAN-2409 + valacyclovir in combination with nivolumab and standard of care in newly diagnosed high-grade glioma. J Immunother Cancer. (2022) 10:A1–A1603. doi: 10.1136/jitc-2022-SITC2022.1477

[195] Hoshida Y Nijman SM Kobayashi M Chan JA Brunet JP Chiang DY. Integrative transcriptome analysis reveals common molecular subclasses of human hepatocellular carcinoma. Cancer Res. (2009) 69:7385–92. doi: 10.1158/0008-5472.CAN-09-1089, PMID: 19723656 PMC3549578

[196] Hernando-Calvo A Vila-Casadesús M Bareche Y Gonzalez-Medina A Abbas-Aghababazadeh F Lo Giacco D. A pan-cancer clinical platform to predict immunotherapy outcomes and prioritize immuno-oncology combinations in early-phase trials. Med. (2023) 4:710–27.e5. doi: 10.1016/j.medj.2023.07.006, PMID: 37572657

[197] Gutierrez M Lam WS Hellmann MD Gubens MA Aggarwal C Tan DSW. Biomarker-directed, pembrolizumab-based combination therapy in non-small cell lung cancer: phase 2 KEYNOTE-495/KeyImPaCT trial interim results. Nat Med. (2023) 29:1718–27. doi: 10.1038/s41591-023-02385-6, PMID: 37429923

[198] Lebofsky R Decraene C Bernard V Kamal M Blin A Leroy Q. Circulating tumor DNA as a non-invasive substitute to metastasis biopsy for tumor genotyping and personalized medicine in a prospective trial across all tumor types. Mol Oncol. (2015) 9:783–90. doi: 10.1016/j.molonc.2014.12.003, PMID: 25579085 PMC5528781

[199] Lu MM Yang Y. Exosomal PD-L1 in cancer and other fields: recent advances and perspectives. Front Immunol. (2024) 15:1395332. doi: 10.3389/fimmu.2024.1395332, PMID: 38726017 PMC11079227

[200] Morrissey SM Yan J. Exosomal PD-L1: roles in tumor progression and immunotherapy. Trends Cancer. (2020) 6:550–8. doi: 10.1016/j.trecan.2020.03.002, PMID: 32610067 PMC7330176

[201] Zhang Y Lee RY Tan CW Guo X Yim WW Lim JC. Spatial omics techniques and data analysis for cancer immunotherapy applications. Curr Opin Biotechnol. (2024) 87:103111. doi: 10.1016/j.copbio.2024.103111, PMID: 38520821

[202] Phillips D Schürch CM Khodadoust MS Kim YH Nolan GP Jiang S. Highly multiplexed phenotyping of immunoregulatory proteins in the tumor microenvironment by CODEX tissue imaging. Front Immunol. (2021) 12:687673. doi: 10.3389/fimmu.2021.687673, PMID: 34093591 PMC8170307

[203] Rivest F Eroglu D Pelz B Kowal J Kehren A Navikas V. Fully automated sequential immunofluorescence (seqIF) for hyperplex spatial proteomics. Sci Rep. (2023) 13:16994. doi: 10.1038/s41598-023-43435-w, PMID: 37813886 PMC10562446

[204] Hernando-Calvo A Garralda E. Patient-centric approaches for phase I combination trials come on stage. Cancer Discov. (2023) 13:1762–4. doi: 10.1158/2159-8290.CD-23-0534, PMID: 37269291 PMC10401070

[205] Avanzo M Wei L Stancanello J Vallières M Rao A Morin O. Machine and deep learning methods for radiomics. Med Phys. (2020) 47:e185–202. doi: 10.1002/mp.13678, PMID: 32418336 PMC8965689

[206] Yousefi Y Baines KJ Maleki Vareki S. Microbiome bacterial influencers of host immunity and response to immunotherapy. Cell Rep Med. (2024) 5:101487. doi: 10.1016/j.xcrm.2024.101487, PMID: 38547865 PMC11031383

[207] Voigt AY Walter A Young TH Graham JP Batista Bittencourt BM de Mingo Pulido A. Microbiome modulates immunotherapy response in cutaneous squamous cell carcinoma. Exp Dermatol. (2023) 32:1624–32. doi: 10.1111/exd.14864, PMID: 37350109 PMC10592435

[208] Glitza IC Seo YD Spencer CN Wortman JR Burton EM Alayli FA. Randomized placebo-controlled, biomarker-stratified phase Ib microbiome modulation in melanoma: impact of antibiotic preconditioning on microbiome and immunity. Cancer Discov. (2024) 14:1161–75. doi: 10.1158/2159-8290.CD-24-0066, PMID: 38588588 PMC11215408

[209] Yildirim Z Swanson K Wu X Zou J Wu J. Next-gen therapeutics: pioneering drug discovery with iPSCs, genomics, AI, and clinical trials in a dish. Annu Rev Pharmacol Toxicol. (2025) 65:71–90. doi: 10.1146/annurev-pharmtox-022724-095035, PMID: 39284102 PMC12011342

[210] Prelaj A Miskovic V Zanitti M Trovo F Genova C Viscardi G. Artificial intelligence for predictive biomarker discovery in immuno-oncology: a systematic review. Ann Oncol. (2024) 35:29–65. doi: 10.1016/j.annonc.2023.10.125, PMID: 37879443

[211] Yoo SK Fitzgerald CW Cho BA Fitzgerald BG Han C Koh ES. Prediction of checkpoint inhibitor immunotherapy efficacy for cancer using routine blood tests and clinical data. Nat Med. (2025) 31:869–80. doi: 10.1038/s41591-024-03398-5, PMID: 39762425 PMC11922749

[212] Arango-Argoty G Bikiel DE Sun GJ Kipkogei E Smith KM Carrasco Pro S. AI-driven predictive biomarker discovery with contrastive learning to improve clinical trial outcomes. Cancer Cell. (2025) 43:761–74. doi: 10.1016/j.ccell.2025.03.029, PMID: 40250446

[213] Friends of Cancer Research. Integrating change in ctDNA levels in advanced cancer clinical trials to support meta-analyses for intermediate endpoint validation(2024). Available online at: https://friendsofcancerresearch.org/ (Accessed May 10, 2025).

[214] U.S. Food and Drug Administration. Use of circulating tumor DNA for early-stage solid tumor drug development: guidance for industry(2022). Available online at: https://www.fda.gov/media/158072/download (Accessed May 10, 2025).

[215] Service description, conformity assessment procedures according to Regulation (EU) 2017/746 on *in-vitro* diagnostic medical devices (IVDR)(2017). Available online at: https://www.tuvsud.com/en/industries/medical-devices/in-vitro-diagnostic-regulation (Accessed Aug 25, 2025).

