# Supplementary material for: Optimizing early-phase immunotherapy trials: the role of biomarker enrichment strategies
Source: Front Immunol. 2025 Oct 22;16:1664443. doi: 10.3389/fimmu.2025.1664443 (PMC12586187; doi:10.3389/fimmu.2025.1664443)
Supplement: Supplementary file 1 [file Table1.docx]

**Supplementary Material**

**Table S1. Concordance/discordance between dMMR/MSI-High testing methods**

| **Method** | **Advantages** | **Limitations** | **Concordance** |
| --- | --- | --- | --- |
| **IHC (MLH1, MSH2, MSH6, PMS2)** | Widely available, low cost, fast TAT | False negatives with specific missense mutations | Discordance 1–10% vs PCR/NGS^46^ |
| **PCR-based MSI** | High sensitivity; reference method | Limited loci | Concordance ~98% with NGS^45^ |
| **NGS panels** | Detects atypical MSI, co-occurring *POLE/POLD* mutations; broad genomic profiling | Higher cost, longer TAT | -~98–99% concordance with PCR  -Discordant in rare atypical cases^47, 48^ |

IHC: immunohistochemistry, PCR: polymerase chain reaction, NGS: next-generation sequencing, TAT: Turnaround Time.

**Table S2. Selected assays for TMB determination**

| **Platform** | **Type** | **Panel size** | **Notes / Limitations** |
| --- | --- | --- | --- |
| **FoundationOne CDx** | NGS targeted panel | 324 genes (~0.8 Mb) | FDA-approved companion diagnostic with ≥10 mut/Mb cutoff^19^ |
| **MSK-IMPACT** | NGS targeted panel | 468 genes (~1.2 Mb) | -Used in >10,000 tumors.  -Strong evidence^72^ |
| **WES (whole exome sequencing)** | NGS | ~30–35 Mb | Gold standard; not widely used clinically due to costs/TAT^49, 50^ |
| **bTMB (e.g., GuardantOMNI and FoundationOne®Liquid CDx)** | ctDNA NGS panels | Variable depending on the panel | Less invasive; 25–30% failure due to low ctDNA; moderate concordance with tTMB (50–60%)^75, 76^ |

NGS: next-generation sequencing, Mb: megabase, TAT: Turnaround Time, tTMB: tissue-based TMB, bTMB: blood TMB, ctDNA: circulating tumor DNA
